# Inhalable Artificial Polymeric Nucleases Degrading Neutrophil Extracellular Trap‐DNAs and Alleviating Pulmonary Fibrosis

**DOI:** 10.1002/advs.202505357

**Published:** 2025-06-20

**Authors:** Yibo Du, Chenxu Zhu, Ruifeng Wang, Shi Chen, Chuang Li, Defang OuYang, Lixin Liu, Yongming Chen

**Affiliations:** ^1^ School of Materials Science and Engineering Key Laboratory for Polymeric Composite and Functional Materials of Ministry of Education Sun Yat‐sen University Guangzhou 510275 China; ^2^ State Key Laboratory of Quality Research in Chinese Medicine Institute of Chinese Medical Sciences (ICMS) University of Macau Macao 999078 China; ^3^ College of Chemistry and Molecular Science Henan University Kaifeng 475004 China; ^4^ State Key Laboratory of Antiviral Drugs Henan University Zhengzhou 450046 China; ^5^ Faculty of Health Sciences University of Macau Macau 999078 China

**Keywords:** artificial DNase, block copolymers, DNA degradation, neutrophil extracellular traps (NETs), pulmonary fibrosis

## Abstract

Pulmonary fibrosis resulting from recurrent lung inflammation due to pathogen infection may lead to serious problems and death. Neutrophil extracellular traps (NETs), consisting of DNAs and proteins released by neutrophils in response to infection, are major pathogenesis factors for pathogen‐associated pulmonary fibrosis. By mimicking nucleic acid hydrolase, polymeric artificial DNases bearing imidazole units (PEG‐PIm) are developed to degrade the DNAs and thus deconstruct NETs, inhibiting pulmonary fibrosis. By tailoring the PIm segments with varied imidazole units, the polymer hydrolase with a defined number of imidazole units outperforms other samples in the cleavage of DNAs and inhibits the transition of pulmonary fibroblasts to myofibroblasts. This polymer digests the DNAs complexed with cationic peptides, unlike natural DNase I. By aerosol inhalation, it reduces NET infiltration in lungs and significantly alleviates inflammatory cytokines and fibrosis. Molecular dynamics simulations indicate that the optimized polymer may expose more effective imidazole units to the DNA backbones and thus enhance the affinity and hydrolysis of phosphodiester linkages. The function is also confirmed by systematic administration of PEG‐PIm to rheumatoid arthritis. Thus, a strategy is provided for treating pulmonary fibrosis that can be applied in a pandemic to reduce high mortality because of pathogen infection.

## Graphical abstract

Pulmonary fibrosis (PF) is promoted by NETs that induce fibroblast activation. PEG‐PIm, a polymeric artificial DNase is reported that efficiently degrades NET‐DNA via phosphodiester bond hydrolysis. Inhalation of PEG‐PIm in a mouse PF model prevents fibroblast activation and fibrosis progression, offering a promising therapeutic strategy for NET‐associated pulmonary fibrosis.

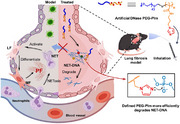

## 1 Introduction

Pulmonary fibrosis (PF) is observed in the late stage of respiratory infection by some pathogens. In the pandemic of COVID‐19, it was reported that ≈20% of patients in 2020 developed PF.^[^ ^1^ ^]^ Some of them progress to hypoxemia, which ultimately results in respiratory failure or even death.^[^ ^2^ ^]^ Currently, the anti‐fibrotic drugs used in the clinical treatment of COVID‐19‐post‐PF include nintedanib, pirfenidone, and corticosteroids. However, their therapeutic effectiveness is limited due to the insufficient anti‐inflammation^[^ ^3^ ^]^ and also serious side effects.^[^ ^4^ ^]^ More potent therapies are required to effectively treat pathogen‐induced PF to reduce infection severity.

A few studies have reported that the levels of neutrophil extracellular traps (NETs) are elevated in the patients of COVID‐19 and are closely associated with poor prognosis.^[^ ^5^ ^]^ The abnormally high NETs in response to infection, composed of neutrophil DNAs and proteins like histones and myeloperoxidases, exacerbate lung injury and promote the transition of lung fibroblasts (LFs) to myofibroblasts (MFs). The proliferated MFs can be further activated by NETs to over‐express α‐smooth muscle actin (α‐SMA) and extracellular matrix (ECM) proteins, causing pulmonary fibrosis.^[^ ^6^ ^]^ Meanwhile, the DNAs of NETs may activate Toll‐like receptor 9 (TLR9) of immune cells as damage‐associated molecular patterns (DAMPs). In patients with idiopathic pulmonary fibrosis (IPF), the TLR9s, which normally appear in the immune cells of healthy individuals, were detected in pulmonary epithelial and smooth muscle cells.^[^ ^7^ ^]^ These activated TLR9s may express cytokine C C motif ligand 2 (CCL2) to promote lung fibrosis, deteriorating the process.

**Figure 1:**
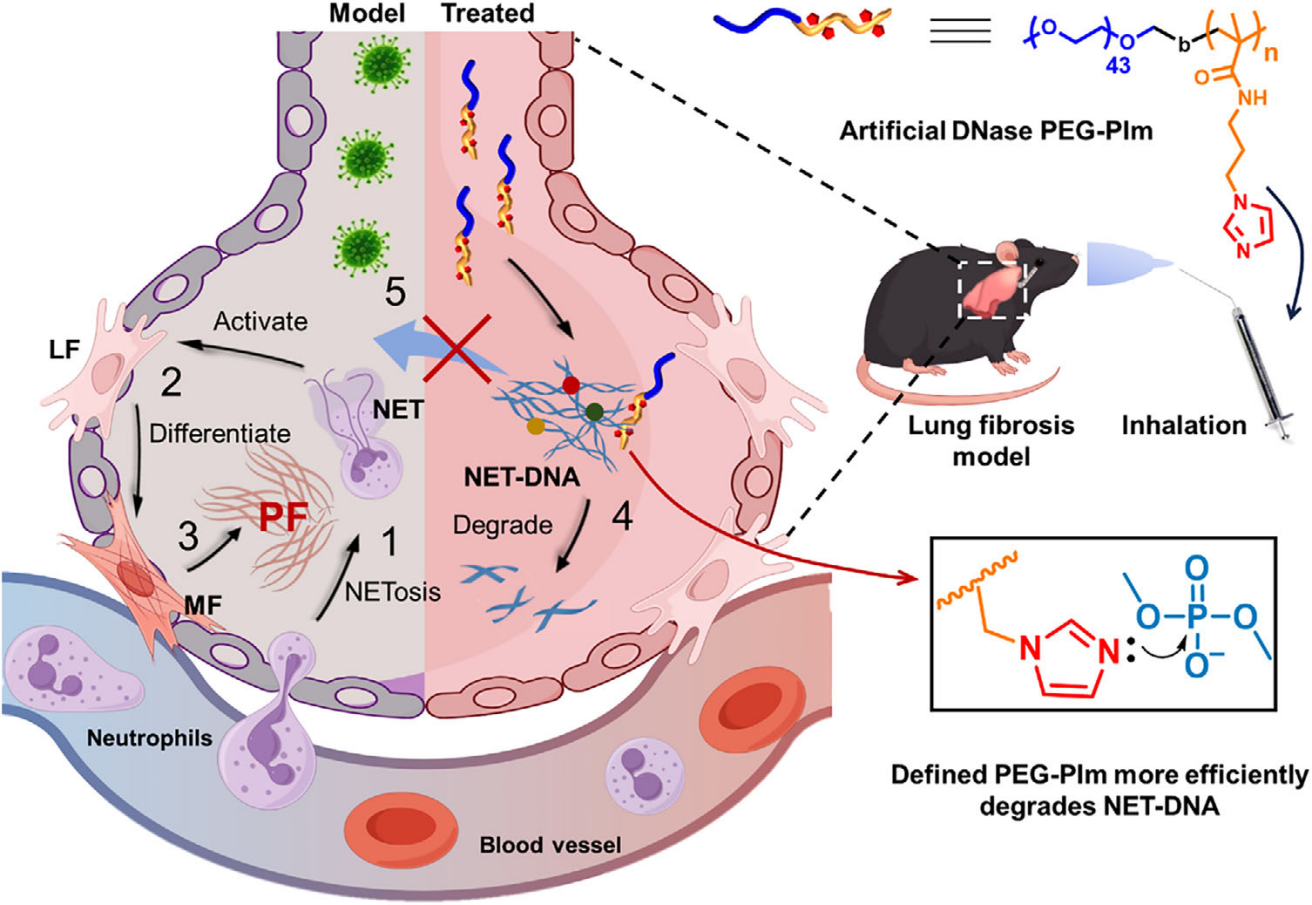
Mechanism of NET clearance and inhibition of NET‐mediated pulmonary fibrosis by PEG‐PIm polymer artificial DNase. 1) Neutrophiles are recruited to pulmonary alveoli because of pathogen infection and release their DNAs and proteins to form neutrophil extracellular traps (NETs), cross‐linked by the components through physical interaction. 2) NETs activate lung fibroblasts (LFs) which are transited and differentiated into myofibroblasts (MFs). 3) Extracellular matrix (ECM) proteins like collagens are expressed from MFs with α‐smooth muscle actin (α‐SMA) as a marker and pulmonary fibrosis (PF) is developed. Expression of Toll‐like receptors (TLRs) in pulmonary epithelial and smooth muscle cells further deteriorates PF. 4) Inhalation of polymeric artificial DNase, PEG‐PIm, catalyzes the hydrolytic cleavage of phosphodiester linkages of DNAs in NETs, 5) thereby preventing NET‐induced transition of primary lung fibroblasts to myofibroblasts, blocking activation of TLRs, and inhibiting pulmonary fibrosis progression. Some elements were produced by www.figdraw.com.

NETs are a crosslinked network of extracellular DNAs and proteins by charge interaction, hydrogen bonding and hydrophobic interaction. We presume that degrading the DNAs in NETs could de‐assemble the NET structure to prevent inflammation and inhibit PF, meanwhile blocking TLR9 activation. Native deoxyribonuclease has already been approved for clinical trial in patients with cystic fibrosis, and several studies have attempted to improve its stability and prolong its half‐life in vivo to enhance the therapeutic effect.^[^ ^8^ ^]^ However, the DNAs in NETs are complexed by proteins, which dampen the hydrolysis efficiency of DNase. To address the limitations of natural DNase, herein we developed a series of poly(ethylene glycol) (PEG)‐*block*‐poly(imidazolyl acrylamide) (PIm) copolymers, PEG‐PIms, with fixed PEG but varied number of imidazole units as artificial DNases to degrade the NET‐DNAs by mimicking imidazole role in natural enzymes in degrading DNA phosphodiester bonds. Compared to natural enzymes, the polymeric artificial nucleic acid enzymes are more stable against hydrolysis and also show potent capacity to cleave DNAs as given in this work. Notably, when they were administered to the pulmonary fibrosis mice model via aerosol inhalation, the PEG‐PIm with a defined number of imidazole units significantly reduced NET infiltration in the lungs and decreased their concentrations in bronchoalveolar lavage fluid (BALF) (**Figure** **1**). Accordingly, the transition of lung fibroblasts to myofibroblasts, level of inflammatory cytokines, and formation of fibrosis in lung were all inhibited by treatment. Moreover, molecular dynamics simulations indicate that the outperformance of the PEG‐PIm is attributed to its more effective imidazole units for the hydrolysis of DNA backbones. This work may supply an alternative strategy to inhibit PF formation induced by pathogen infection and alleviate the symptoms thereof.

## 2 Results

### 2.1 PEG‐PIm_10_ Efficiently Hydrolyzes Nucleic Acids

Imidazole units in DNases are responsible for cleaving phosphodiester linkage between nucleic bases and the polymers bearing imidazole units have been applied as artificial DNase to cleave the nucleic acids in vitro and in vivo.^[^ ^9^ ^]^ We applied N‐imidazole‐3‐propylmethacrylamide as monomers to subject controlled radical polymerization in order to obtain the polymers with a well‐defined composition. To improve the solubility in water and biocompatibility of the materials in vivo, we used PEG of 2 K Dalton with one terminal functionalized with a chain transfer agent (CTA) for the reversible addition‐fragmentation chain transfer (RAFT) polymerization. A series of block copolymers, PEG‐PIm_n_ (*n* = 5, 10, 30, and 40), with varied degrees of polymerization of PIm but fixed PEG, were obtained in order to evaluate the catalytic properties of polymeric artificial nuclease (Synthesis and characterization were collected in Figures S1–S5 and Table S1, Supporting Information). In the pH 7.4 PBS buffer, four polymers formed nanoparticles of diameters ≈100 nm, indicating they are amphiphilic in nature. Zeta potentials of them showed positively charged in general (Figure S6 and Table S2, Supporting Information). This is expected since imidazole is a weak base and thus its polymers may be partially protonated at pH 7.4. The relatively higher zeta potential of PEG‐PIm_10_ implies its more imidazole units being exposed on the surface of particles. It is worth noting that all four PEG‐PIm polymers exhibit good biocompatibility (Figure S7A, Supporting Information).

**Figure 2:**
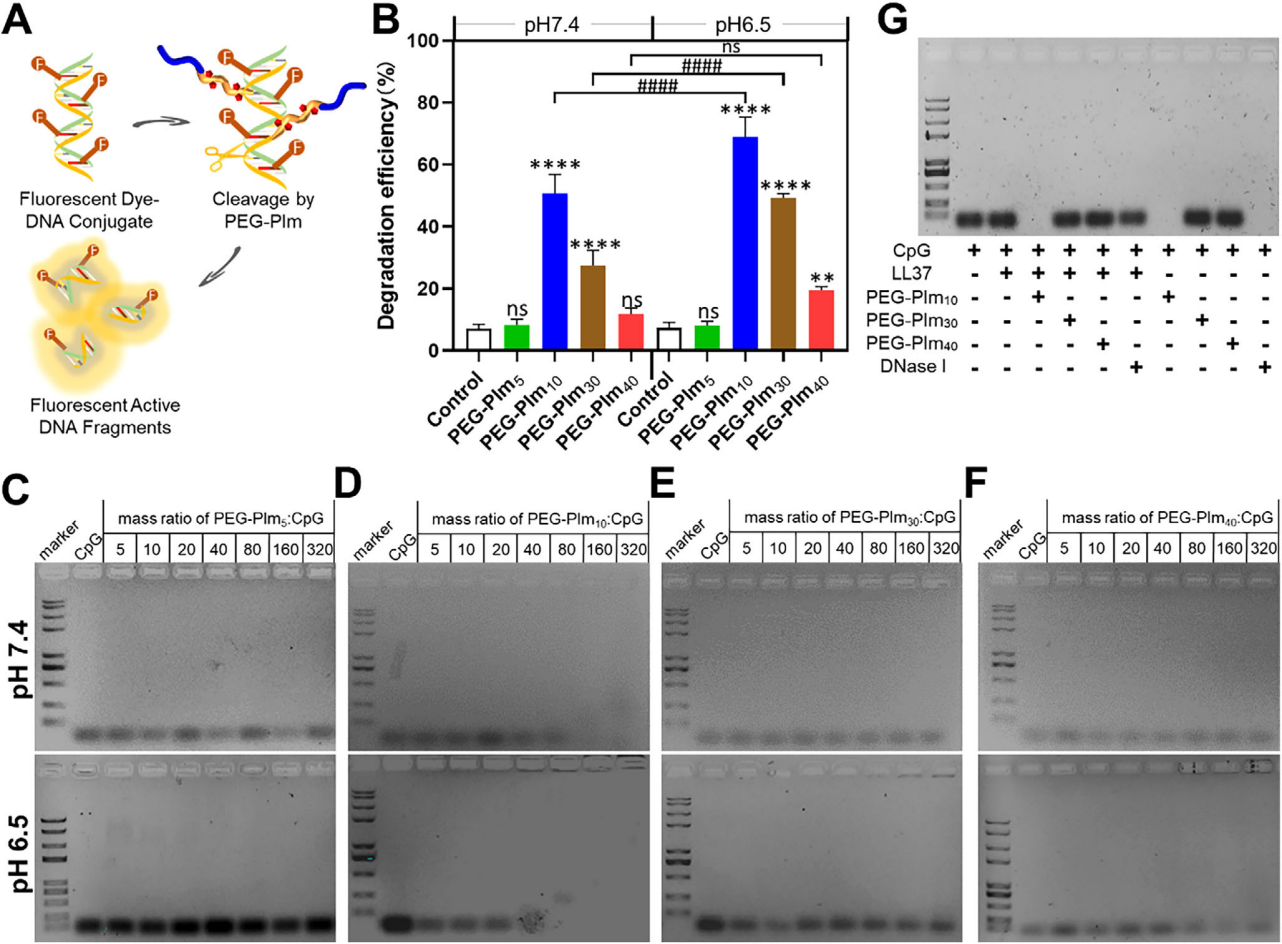
The degradation efficiency of nucleic acids by PEG‐PIm treatment. A) Principle of the DNase I Assay Kit used to evaluate PEG‐PIm‐mediated DNA hydrolysis. B) DNA degradation efficiency (DNA fragments (pmol) / DNA probe complete degradation (pmol) × 100%) of PEG‐PIm as measured by the DNase I activity detection kit at pH 7.4 (left) and pH 6.5 (right) for 6 h (*n* = 3, mean ± S.E.M.; ^*^0.01 < *p* < 0.05, ^**^0.001 < *p* < 0.01, ^***^0.0001 < *p* < 0.001 compared to control; ^#^0.01 < *p* < 0.05, ^##^0.001 < *p* < 0.01, ^###^ *p* < 0.001 between groups). C–F) Agarose gel electrophoresis of CpG hydrolyzed by PEG‐PIm in PBS at pH 7.4 and 6.5. G) Agarose gel electrophoresis for CpG degradation by PEG‐PIm treatment (polymer/CpG = 80/1) in the presence of cationic LL37 at pH 7.4. The agarose gels were stained with Gene Green NA dye.

The cleavage capabilities of PEG‐PIm_n_ to nucleic acids were assessed through a DNase Assay Kit containing a DNA probe, which allows for quantitative evaluation of the hydrolysis activity by detecting the fluorescent DNA fragments (**Figure** **2**A). Under physiological conditions (pH 7.4) and polymer‐to‐DNA ratio of 80/1, while PEG‐PIm_5_ nearly had no function, PEG‐PIm_10_, PEG‐PIm_30_, and PEG‐PIm_40_ in 6 h exhibited hydrolysis efficiency of 56.2%, 31.6%, and 13.4%, respectively (Figure 2B). Contrary to our expectations, the catalytic efficacy decreased when the number of imidazole repeating units in the polymers increased from 10 to 40. To further confirm this phenomenon, four samples were incubated with CpG ODN 1826, oligo single‐stranded DNA, as the model DNA at pH 7.4 and then their degraded fragments were analyzed with agarose gel electrophoresis. PEG‐PIm_10_ completely degraded CpG at an 80:1 polymer‐to‐CpG mass ratio (Figure 2C–F). However, PEG‐PIm_5_, PEG‐PIm_30,_ and PEG‐PIm_40_ could not hydrolyze CpG even at a polymer‐to‐CpG mass ratio as high as 320:1, consistent with the above results. Considering the microenvironment of the fibrotic lung was weak and acidic, we further investigated the DNA degradation ability of PEG‐PIms at pH 6.5. While PEG‐PIm_5_ still did not work, PEG‐PIm_10_, PEG‐PIm_30_, and PEG‐PIm_40_ exhibited degradation efficiency of 68.9%, 49.7%, and 19.5%, respectively (Figure 2B). Among them, PEG‐PIm_10_ still showed the highest activity to cleave the DNA. The results were also supported by cleaving CpG at pH 6.5 using the agarose gel electrophoresis (Figure 2C–F). Notably, the cleaving activity at an acidic condition showed some increasement relative to that at pH 7.4.

As NETs are composed of DNAs and proteins released from neutrophils, we further evaluated the degradation of CpG in the presence of cationic peptide LL37 as model proteins, taking natural nucleases (DNase I) as control. As shown in Figure 2G, PEG‐PIm_10_ digested CpG no matter with or without LL37 at a ratio of polymer‐to‐CpG of 80. However, PEG‐PIm_30_ and PEG‐PIm_40_ showed much lower hydrolysis activity to CpG with LL37. It is interesting to notice that, though DNase I may digest free CpG completely, it showed much lower capability to hydrolyze CpG in the presence of LL37. Thus, PEG‐PIm_10_ had a much better capacity to cleave the nucleic acids in the protein complexes than the DNase. The imidazole units along polymer chains may be partially protonated at physical conditions, which is needed in hydrolysis reaction. Thus, their binding ability to negative DNA was evaluated in phosphate‐buffered saline (PBS) and 10% fetal bovine serum (FBS) in the presence of ethidium bromide (EtBr). Especially at higher mass ratio of polymer‐to‐DNA, four samples showed competitive binding capacities and PEG‐PIm_10_ outperformed in either PBS or FBS (Figure S8, Supporting Information). This property may correlate with the hydrolysis properties above and inhibition below.

### 2.2 PEG‐PIm_10_ Inhibits NETs‐Induced Migration of Primary Lung Fibroblasts and Transition to Myofibroblasts

**Figure 3:**
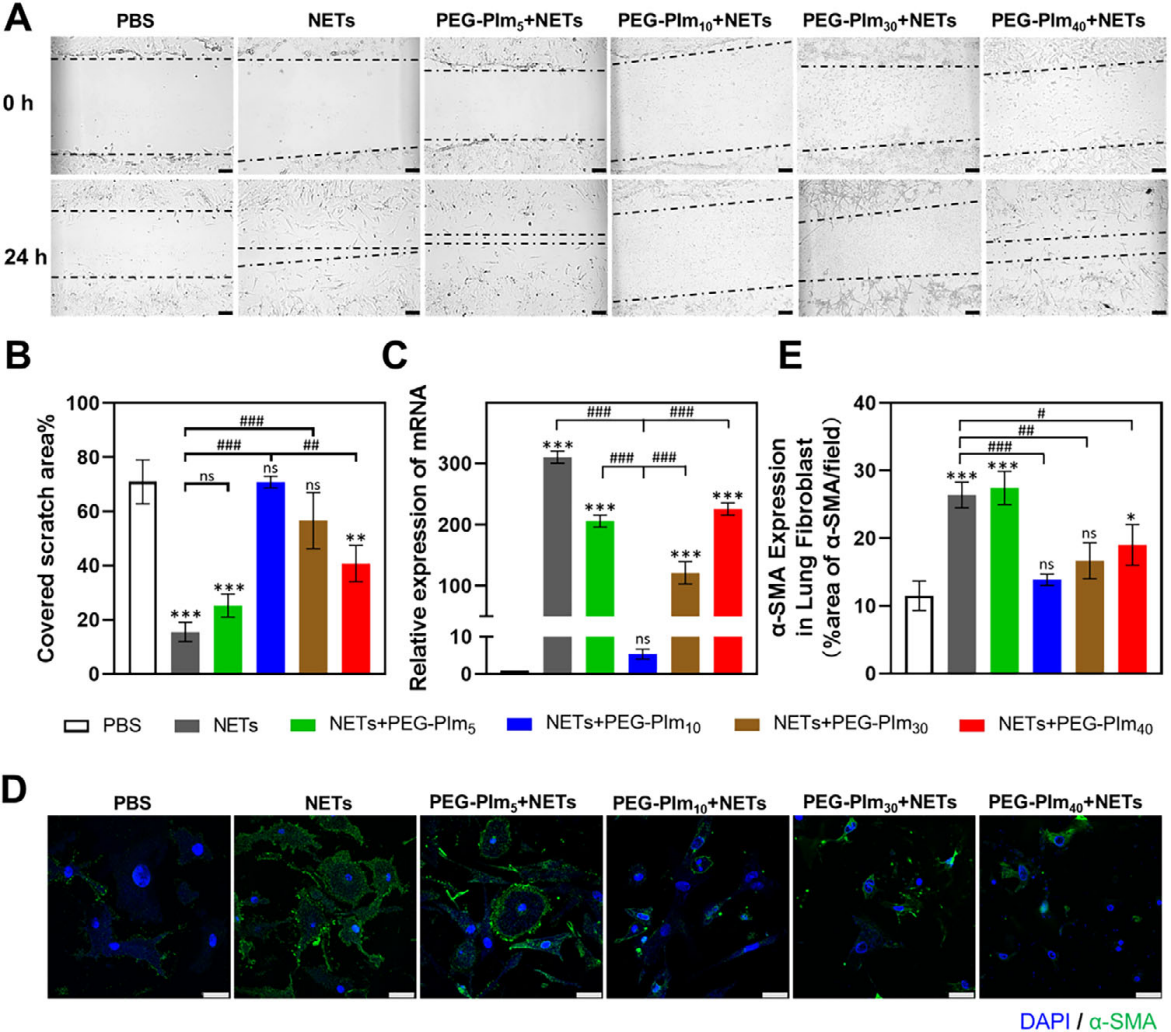
Inhibition of transition of lung fibroblasts to myofibroblasts mediated by NETs with PEG‐PIms. A) Images of cell migration at the same position of primary lung fibroblasts from C57 mice treated with NETs and four PEG‐PIm samples for 24 h, scale bar: 100 µm. B) Quantification of the percentage of cell coverage in the blank area. *n* = 3, mean ± S.E.M.; ^*^0.01 < *p* < 0.05, ^**^0.001 < *p* < 0.01, ^***^0.0001 < *p* < 0.001, compared to the PBS group; ^#^ 0.01 < *p* < 0.05, ^##^ 0.001 < *p* < 0.01, ^###^0.0001 < *p* < 0.001 between groups. C) mRNA expression level of α‐SMA in primary lung fibroblasts co‐treated with PEG‐PIm samples and NETs. *n* = 3, mean ± S.E.M.; ^*^0.01 < *p* < 0.05, ^**^0.001 < *p* < 0.01, ^***^0.0001 < *p* < 0.001 compared to the PBS group; ^#^ 0.01 < *p* < 0.05, ^##^ 0.001 < *p* < 0.01, ^###^0.0001 < *p* < 0.001 between groups. D) Laser confocal microscopy images of α‐SMA protein secretion from primary lung fibroblasts treated with NETs and PEG‐PIm, scale bar: 25 µm. E) Quantitative analysis of (D). *n* = 3, mean ± S.E.M.; ^*^0.01 < *p* < 0.05, ^**^0.001 < *p* < 0.01, ^***^0.0001 < *p* < 0.001 compared to the PBS group; ^#^ 0.01 < *p* < 0.05, ^##^ 0.001 < *p* < 0.01, ^###^0.0001 < *p* < 0.001 between groups.

Pulmonary fibrosis begins with abnormal activation of primary lung fibroblasts (LFs), followed by their migration, proliferation, and differentiation into myofibroblasts. NETs can induce the procedure occurrence and promote pulmonary fibrosis.^[^ ^6^, ^10^ ^]^ As PEG‐PIm_10_ could efficiently digest nucleic acids complexed with protein, we evaluated whether it could inhibit NET‐induced migration of primary lung fibroblasts by disruption of NETs structure via scratch experiments.^[^ ^6^ ^]^ The scratch area was evaluated after incubation of LFs with NETs in the presence of PEG‐PIm, taking PBS or NETs as control. As shown in **Figure** **3**A,B, NETs induced migration of LFs as given by the scratch area reduced to 15.5% of the original area. In the meanwhile, the scratch area of NETs with PEG‐PIm_10_ was almost same as that of PBS (70.8% vs 72%), indicating that the migration of LFs induced by NETs was significantly inhibited by PEG‐PIm_10_. In contrast, the scratch area of PEG‐PIm_5_, PEG‐PIm_30_, and PEG‐PIm_40_ were 25.3%, 56.6%, and 40.8% of the original area, respectively. These results matched the cleaving capacities of the artificial DNase. Next, we evaluated whether PEG‐PIms could inhibit the transition of LFs to myofibroblasts, which could express more α‐SMA and deposit more collagen matrix to exacerbate pulmonary fibrosis. After incubation of LFs with NETs together with four samples for 24 h, the mRNA level of α‐SMA was quantitated (Figure 3C). PEG‐PIm_10_ showed the best inhibitory effect, and α‐SMA decreased nearly 30‐fold relative to that of the NET group. The results were further verified by immunostaining assay with fluorescence‐labeled α‐SMA antibodies (Figure 3D,E). Compared with LFs in NETs, the presence of PEG‐PIms inhibited α‐SMA expression in lung fibroblast and PEG‐PIm_10_ still showed the best performance. Therefore, NETs‐mediated migration of primary lung fibroblasts and transition to myoblast in vitro could be inhibited by the polymeric DNase on cleaving DNAs.

### 2.3 Inhalation of PEG‐PIm_10_ Significantly Alleviates Pulmonary Fibrosis

**Figure 4:**
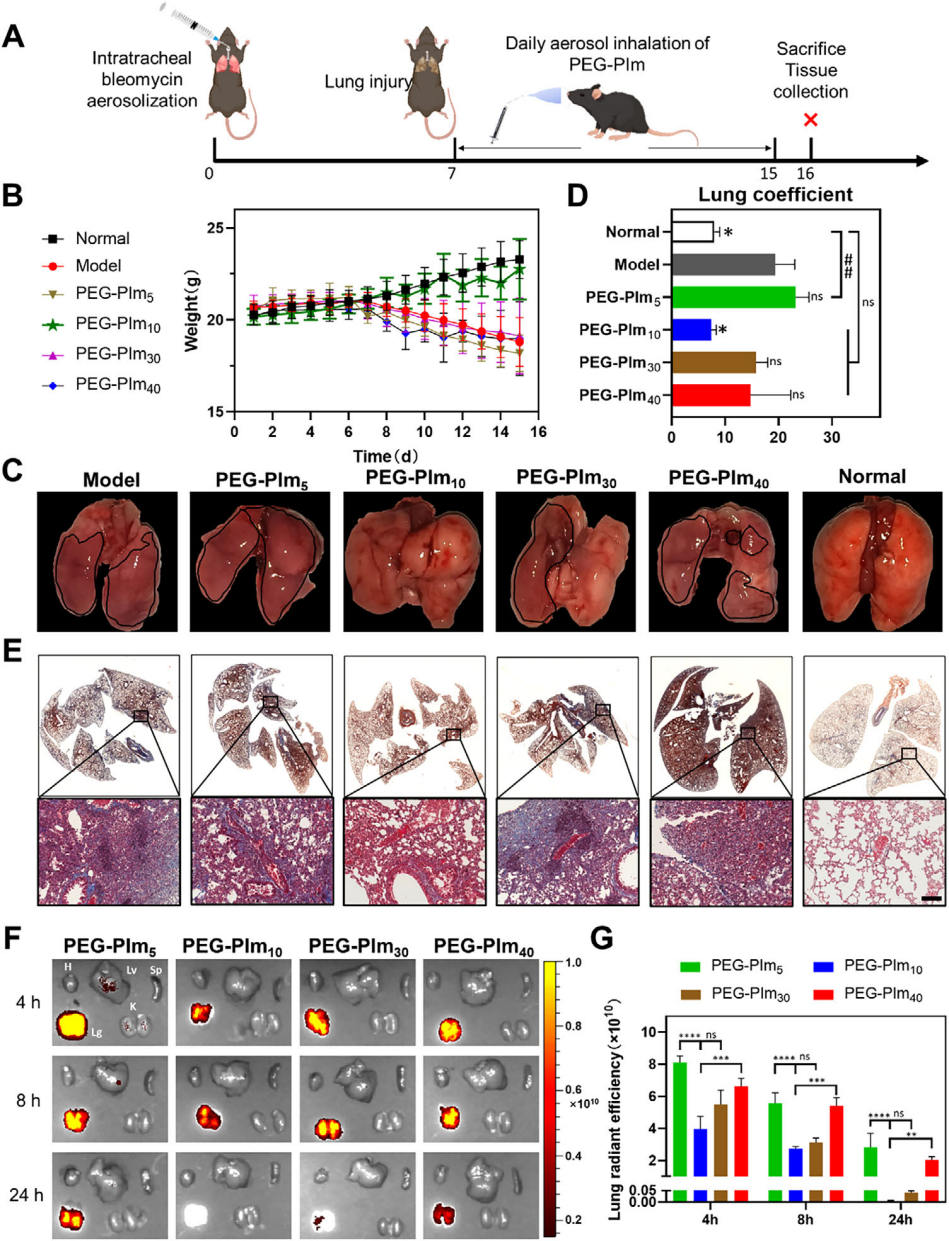
Alleviation of pulmonary fibrosis symptoms in mice models with PEG‐PIm treatment. A) Treatment timeline for mice with pulmonary fibrosis. After intratracheal injection of bleomycin solution, a model of pulmonary fibrosis was induced on day 7. Then mice inhaled a solution aerosol of PEG‐PIm daily. Mice were euthanized on day 16 for further studies. B) Changes in body weight of mice during treatment. C) Anatomical photos of the lungs in treatment groups on day 16. The areas outlined by blacklines indicate visible pulmonary fibrosis symptoms. D) Changes in lung coefficients, the weight ratio of the lung to the body (mg/g), on day 16 in different groups. *n* = 6, mean ± S.E.M.; ^*^0.01 < *p* < 0.05, ^**^0.001 < *p* < 0.01, ^***^0.0001 < *p* < 0.001 compared to the Model group; ^#^ 0.01 < *p* < 0.05, ^##^ 0.001 < *p* < 0.01, ^###^0.0001 < *p* < 0.001 between groups. E) Masson staining histological analysis of lungs, with the blue areas in Masson staining representing collagen deposition, scale bar: 100 µm. F) *Ex vivo* NIRF images of the organs from C57 mice after nebulized inhalation of AF750‐labeled PEG‐PIm at 4, 8, and 24 h. H: heart; Lv: liver; Sp: spleen; Lg: lung; K: kidneys. (G) NIRF intensity of PEG‐PIm in the lungs at different time points. *n* = 3, mean ± S.E.M.; ^*^0.01 < *p* < 0.05, ^**^0.001 < *p* < 0.01, ^***^0.0001 < *p* < 0.001, ^****^ *p* < 0.0001 between groups.

To check the performance in animals, we atomized the aqueous solution of PEG‐PIm into a mouse model of pulmonary fibrosis (PF) induced by bleomycin (BLM) (**Figure** **4**A). Treatment was stopped when the models without any treatment started obvious loss of body weight (Figure 4B). After 8 days of daily treatment, the average weights of the model mice in the control group, PEG‐PIm_5_, PEG‐PIm_10_, PEG‐PIm_30_, PEG‐PIm_40_, and normal groups were 18.8 g, 18.2 g, 22.8 g, 19.1 g 19.0 g, and 23.3 g, respectively. The weight of mice in the PEG‐PIm_10_ group gradually increased to that of normal mice, indicating that the progression of pulmonary fibrosis was effectively controlled. In contrast, the mice weights of other groups decreased, showing that pulmonary fibrosis severely impaired the health of the mice, and the other three polymer groups did not alleviate the symptoms of pulmonary fibrosis.

Then, the lungs of the mice were collected. In contrast to the normal lungs, the fibrotic lungs of model mice became dusky, as indicated by the black lines in Figure 4C. After treatments, the appearance in groups of PEG‐PIm_10_, PEG‐PIm_30,_ and PEG‐PIm_40_ became better. Especially, the lungs of mice in the PEG‐PIm_10_ group were close to that of the normal group. The results were further confirmed by the mouse lung coefficient, which is used to evaluate lung inflammation, edema, and fibrosis. The average lung coefficient of the mice in the PEG‐PIm_10_ group was almost the same as that of the normal mice (7.4 & 7.9), whereas these of PEG‐PIm_5_ (23.1), PEG‐PIm_30_ (15.8), and PEG‐PIm_40_ (14.7) groups were significantly higher than that of normal mice and close to the model mice (19.4) (Figure 4D). Moreover, the extent of pulmonary fibrosis was investigated via Masson's trichrome staining collagen on lung sections. Consistent with the above experiments, compared with model mice, collagen deposition in the lung was significantly reduced in the PEG‐PIm_10_ group, outperforming other samples in treatment (Figure 4E). Thus, PF could be greatly inhibited by the optimized sample.

Since inhalation can deliver the drug directly into the airway, reaching high local concentrations in the lungs and preventing systemic toxicity. We explored the distribution of PEG‐PIms after nebulized inhalation in mice. The *ex vivo* near‐infrared fluorescence (NIRF) imaging showed that all PEG‐PIms remained in the lungs of mice at various time points (Figure 4F). The fluorescence signals in the lungs declined over time according to orders of PEG‐PIm_10_, PEG‐PIm_30_, PEG‐PIm_40,_ and PEG‐PIm_5_ (Figure 4G). It was interesting to note that this order seems to be related to the positively charged polymer materials. The zeta potentials of PEG‐PIm_10_, PEG‐PIm_30_, PEG‐PIm_40,_ and PEG‐PIm_5_ also decreased following the same order (Table S2, Supporting Information). It is noteworthy that no leakage to other internal organs, including the liver and kidneys, was observed, indicating that the PEG‐PIms cannot penetrate into the systemic circulation (Figure 4F). This may benefit the application because fewer side effects could have resulted. It is worth noting that all four PEG‐PIm polymers exhibit low toxicity toward primary lung cells and do not cause local lung injury after inhalation (Figure S7B, Supporting Information).

### 2.4 PEG‐PIm_10_ Effectively Reduces Pulmonary NET Accumulation and Inhibits Inflammation in Lung

**Figure 5:**
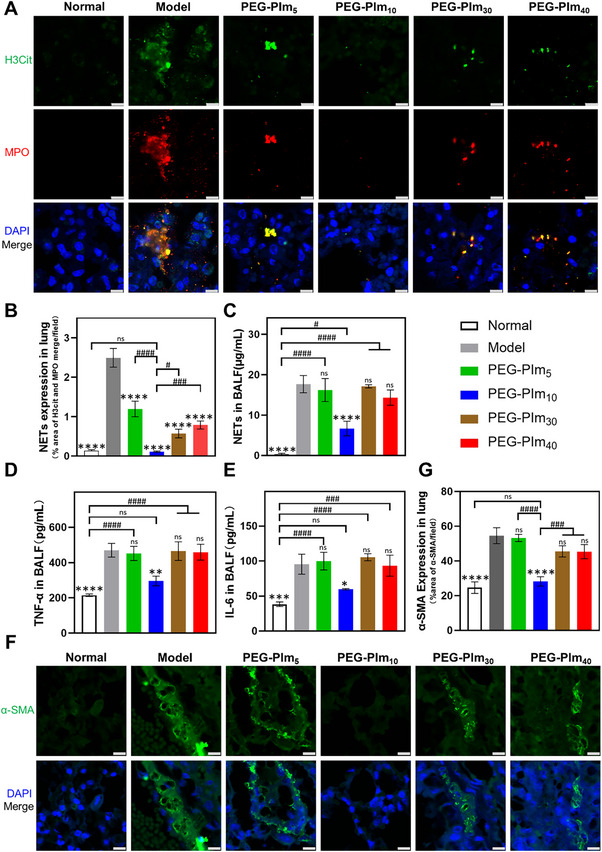
Evaluation of NET infiltration in the lungs of mice with pulmonary fibrosis and inhibition of pulmonary inflammation by treatment with PEG‐PIms. A) Immunostaining images of MPO and H3Cit in NET infiltration in the lungs on day 16, H3Cit (green), MPO (Red), and DNA (Blue). B) Quantification of NET level of (A), statistical analysis using ImageJ. *n* = 3, mean ± S.E.M.; ^*^0.01 < *p* < 0.05, ^**^0.001 < *p* < 0.01, ^***^0.0001 < *p* < 0.001 compared to the model group; ^#^ 0.01 < *p* < 0.05, ^##^ 0.001 < *p* < 0.01, ^###^0.0001 < *p* < 0.001 between groups. C) The concentration of NETs in the bronchoalveolar lavage fluid (BALF) on day 16 of treatment. *n* = 6, mean ± S.E.M.; ^*^0.01 < *p* < 0.05, ^**^0.001 < *p* < 0.01, ^***^0.0001 < *p* < 0.001 compared to the model group; ^#^ 0.01 < *p* < 0.05, ^##^ 0.001 < *p* < 0.01, ^###^0.0001 < *p* < 0.001 between groups. D) Concentration of TNF‐α in the BALF on day 16 of treatment, *n* = 6, mean ± S.E.M.; ^*^0.01 < *p* < 0.05, ^**^0.001 < *p* < 0.01, ^***^0.0001 < *p* < 0.001, ^****^ *p* < 0.0001 compared to the Model group; ^#^0.01 < *p* < 0.05, ^##^0.001 < *p* < 0.01, ^###^0.0001 < *p* < 0.001, ^####^ *p* < 0.0001 between groups. E) Concentration of IL‐6 in the BALF on day 16 of treatment. *n* = 6, mean ± S.E.M.; ^*^0.01 < *p* < 0.05, ^**^0.001 < *p* < 0.01, ^***^0.0001 < *p* < 0.001, ^****^ *p* < 0.0001 compared to the Model group; ^#^0.01 < *p* < 0.05, ^##^0.001 < *p* < 0.01, ^###^0.0001 < *p* < 0.001, ^####^ *p* < 0.0001 between groups. F) Immunostaining images of α‐SMA represented the transition of pulmonary fibroblasts to myofibroblasts. G) Quantification of lung α‐SMA expression, *n* = 3, mean ± S.E.M.; ^*^0.01 < *p* < 0.05, ^**^0.001 < *p* < 0.01, ^***^0.0001 < *p* < 0.001, ^****^ *p* < 0.0001 compared to the Model group; ^#^0.01 < *p* < 0.05, ^##^0.001 < *p* < 0.01, ^###^0.0001 < *p* < 0.001, ^####^ *p* < 0.0001 between groups, scale bar: 8 µm.

Next, we explored whether the therapeutic effects of PEG‐PIms on pulmonary fibrosis were attributed to its destruction of NETs in vivo. After nebulizing PEG‐PIms to mice 8 days, we detected NET levels in the lung sections of mice by immunostaining with myeloperoxidase (MPO) and citrullinated histone H3 (H3Cit) antibodies. Both H3Cit and MPO, main components of NETs, can serve as key markers because MPO is released with DNA, and histone H3 undergoes citrullination to become H3Cit during NETosis. As indicated by H3Cit and MPO, the NETs in PEG‐PIm_5_, PEG‐PIm_10_, PEG‐PIm_30_, and PEG‐PIm_40_ treating groups were reduced to 57%, 2.7%, 40%, and 30% of the model group (**Figure** **5**A,B). Among different samples, PEG‐PIm_10_ showed the most notable reduction of NETs. Furthermore, we evaluated the levels of NETs in the BALF (Figure 5C). The concentration of NETs in the model group (17.7 µg mL^−1^) was significantly higher than that in the normal group (0.32 µg mL^−1^). After PEG‐PIm_10_ treatment, the concentration of NETs (6.68 µg mL^−1^) decreased significantly, while these in PEG‐PIm_5_ (16.2 µg mL^−1^), PEG‐PIm_30_ (17.1 µg mL^−1^) and PEG‐PIm_40_ (14.3 µg mL^−1^) groups did not decrease compared with the model group. It corroborated with the immunofluorescence of NETs in the lung sections after being treated with PEG‐PIms.

Accordingly, the levels of inflammatory cytokines, TNF‐α, and IL‐6, which were induced by NETs, showed a reduction trend as NETs in BALF (Figure 5D,E). In the model group, the levels of TNF‐α, 469.8, and IL‐6, 95.5 pg mL^−1^, increased significantly compared with the healthy mice, TNF‐α 215.0 and IL‐6 38.4 pg mL^−1^. After PEG‐PIm_10_ treatment, the levels of TNF‐α and IL‐6 were significantly decreased to 295.9 and 59.8 pg mL^−1^. Whereas those by PEG‐PIm_5_ (TNF‐α 451.7, IL‐6 99.8 pg mL^−1^), PEG‐PIm_30_ (TNF‐α 465.1, IL‐6 105.5 pg mL^−1^), and PEG‐PIm_40_ (TNF‐α 458.7, IL‐6 93.3 pg mL^−1^) were not effectively reduced. Similar to the destruction of NETs and the inhibition of inflammatory factors, PEG‐PIm_10_ significantly reduced α‐SMA expression in lung sections, almost to the same level as the normal group (Figure 5F,G). Whereas, the other three samples could not effectively inhibit α‐SMA expression. Thus, PEG‐PIm_10_ could significantly reduce lung NET levels, suppress the production of inflammatory factors, and inhibit myofibroblast differentiation, thereby blocking lung fibrosis.

### 2.5 Hydrolysis DNAs by PEG‐PIms Is Influenced by Composition

From the above data, we found that the PEG‐PIm_10_ outperformed in cleavage of DNA backbones and inhibiting of NET‐DNA induced inflammation among four samples, indicating tailored composition is important. It is understandable for the poor performance of PEG‐PIm_5_ is because of fewer imidazole units. However, PEG‐PIm_30_ and PEG‐PIm_40_ also showed less performance than PEG‐PIm_10_, which was unexpected. As given in Table S2 (Supporting Information), these polymers with amphiphilic in nature appeared as nanoparticles with PEG shell and PIm core.

**Figure 6:**
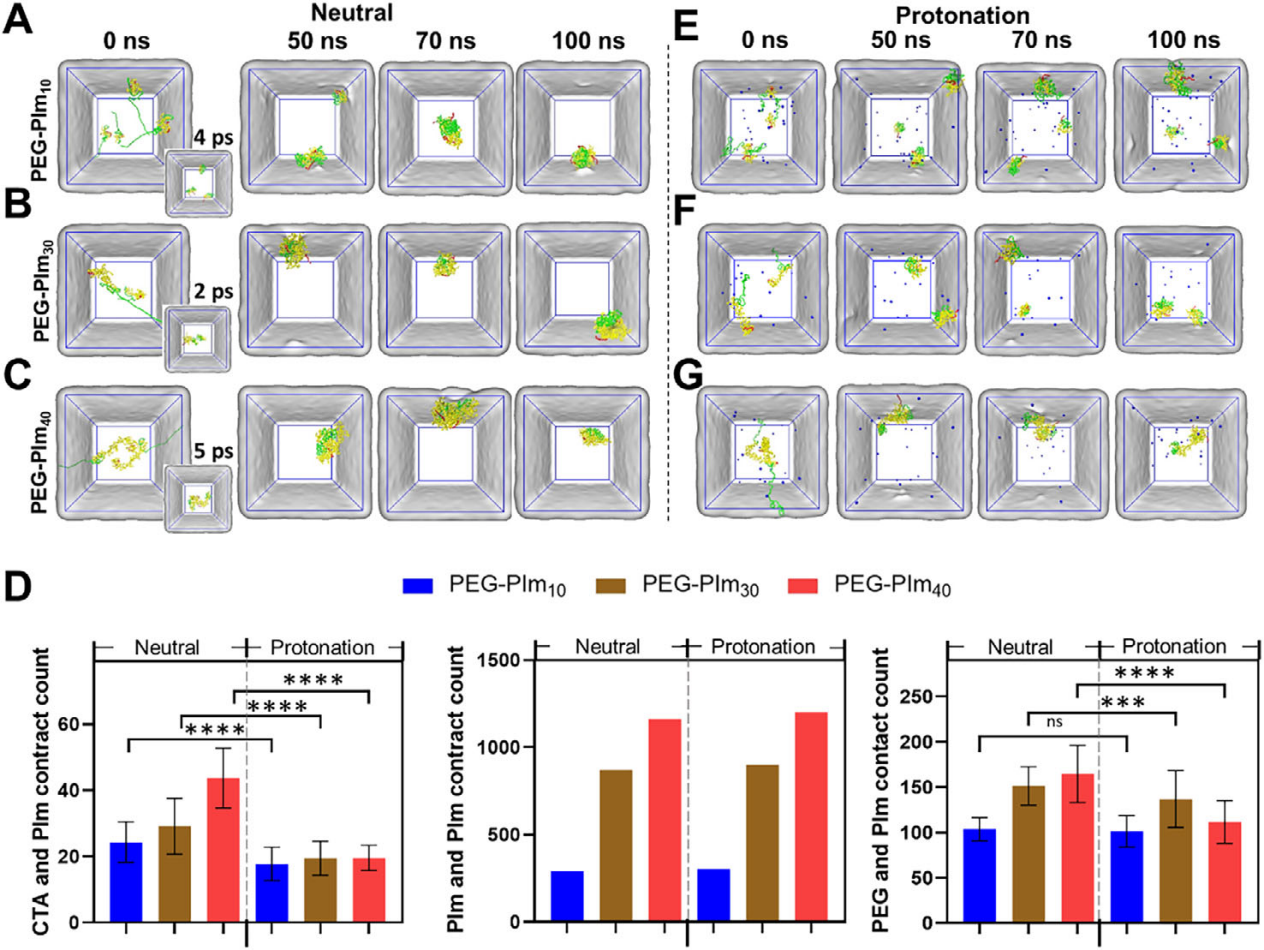
Molecular dynamics simulations reveal the self‐assembly kinetics and molecular conformations of PEG‐PIm with different degrees of polymerization in solution. A–C) Molecular conformations of PEG‐PIm_10_ (A), PEG‐PIm_30_ (B), and PEG‐PIm_40_ (C) after self‐assembly at different time points under neutral conditions. D) The count of contacts between the functional groups of the PIm segment and CTA segment, PIm segment and PIm segment, and PIm segment and PEG segment of the three PEG‐PIms under neutral and protonated conditions (*n* = 101, mean ± S.E.M.; ^*^0.01 < *p* < 0.05, ^**^0.001 < *p* < 0.01, ^***^0.0001 < *p* < 0.001, ^****^ *p* < 0.0001 between groups). E–G) Molecular conformations of PEG‐PIm_10_ (E), PEG‐PIm_30_ (F), and PEG‐PIm_40_ (G) after self‐assembly at different time points under protonated conditions. In the visual field, the light grey represents the aqueous environment; red, yellow, and green represent different segments of the PEG‐PIm polymer, and blue spheres represent chloride ions used to neutralize the positive charges in the system.

The exposing of imidazole units is important for the cleavage of DNA backbones. To elucidate the conformation of PEG‐PIm chains in the micellar particles, molecular dynamic (MD) simulations were conducted at pH 7.4. Based on the molar concentration of imidazole units, PEG‐PIm_10_, PEG‐PIm_30_, and PEG‐PIm_40_ were added to the simulation system in accordance with the actual experiment, as shown in Table S3 (Supporting Information). Simulations aimed to analyze the self‐assembly behavior and conformational dynamics of these polymers in an aqueous solution, which may facilitate the assessment of the polymer segments for interactions. The results showed that PEG‐PIm_10_ molecules took 70 ns to form a nanoparticle, while PEG‐PIm_30_ and PEG‐PIm_40_ can form nanoparticles within 5 ps, much faster than PEG‐PIm_10_ (**Figure** **6**A–C). Thus, the higher the degree of polymerization of PIm is, the higher the self‐assembly ability is. Namely, PEG‐PIm_30_ and PEG‐PIm_40_ are more likely to aggregate into nanoparticles, resulting in more imidazole being wrapped in the particles by PEG shells. Moreover, the instantaneous contact count of different functional groups, PIm and CTA (terminal of PIm), PIm and PIm, PIm and PEG, were analyzed over a period of 100 ns. These contact counts all increased as the degree of polymerization of PIm increases (Figure 6D). The more contacts, the lower the mobility. The PEG segments of PEG‐PIm nanoparticles are oriented externally, acting as hydrophilic barriers that shield the PIm core from direct interaction with the surroundings. The lower contact count between PEG‐PIm indicates less PEG shielding PIm, leaving more imidazole groups exposed to the environment, thus exhibiting high efficiency in the hydrolysis of nucleic acids. Thus, the imidazole in PEG‐PIm_10_ is less shielded, which can explain why PEG‐PIm_10_ has the strongest hydrolysis function to nucleic acids.

Furthermore, the self‐assembly dynamics of polymers were simulated under protonated conditions. In the PEG‐PIm_10_ system, only two molecules in 50 ns aggregated to form a dimer, while the remaining molecules stayed monomerically. For PEG‐PIm_30_, no complete nanoparticle formed throughout the simulation period, whereas PEG‐PIm_40_ formed a nanoparticle within 15 ns, maintaining this structure until the end of the simulation (100 ns) (Figure 6E–G). Thus, among PEG‐PIms, only PEG‐PIm_40_ could form a nanoparticle under protonated conditions, and the time is significantly longer compared to neutral conditions. These findings suggest that the protonation diminishes the self‐assembly capacity of PEG‐PIms, due to increased hydrophilicity of the PIm segments and electrostatic repulsion interaction, which reduces the intermolecular forces driving self‐assembly. Moreover, in an acidic microenvironment, the interaction between PIm and other groups decreased compared with that in neutral conditions. In PEG‐PIm_10_, PEG‐PIm_30_, and PEG‐PIm_40_, the contact count between PEG and PIm decreased compared to neutral conditions. Additionally, a greater reduction in the contact number between PIm and CTA was observed when these polymers were under protonated conditions. However, the contact count between different imidazole groups within the PIm segment remains consistent every nanosecond and does not vary with time. Therefore, the simulation results provide a molecular‐level explanation for the experimentally observed enhancement in hydrolysis efficiency under acidic conditions. Overall, the PIm segment in PEG‐PIm_10_ exhibited fewer contacts with PEG and CTA segments compared to those in PEG‐PIm_30_ and PEG‐PIm_40_ under both acidic and neutral conditions, which accounts for its superior nucleic acid hydrolysis activity.

Moreover, we measured the critical micelle concentrations (CMC) of the four PEG‐PIm polymers (Figure S9, Supporting Information). A lower CMC indicates a stronger self‐assembly driving force. Under neutral conditions (pH 7.4), the CMC values of PEG‐PIm_5_, PEG‐PIm_10_, PEG‐PIm_30_, and PEG‐PIm_40_ were 3.20, 2.47, 1.13, and 0.72 mg mL^−1^, respectively. As the number of imidazole units increased, the self‐assembly capability of the polymers gradually improved. Under acidic conditions (pH 6.5), PEG‐PIm_5_ and PEG‐PIm_10_ did not self‐assemble within the tested concentration range, while the CMCs of PEG‐PIm_30_ and PEG‐PIm_40_ increased to 2.80 and 2.49 mg mL^−1^, respectively. These results support the findings of our molecular dynamics simulations, highlighting the impact of PEG‐PIm segment length on self‐assembly behavior and further elucidating the underlying reason for the differences in nucleic acid hydrolysis efficiency among the PEG‐PIm variants.

### 2.6 PEG‐PIm_10_ Effectively Degrades DNAs via Systemic Administration

**Figure 7:**
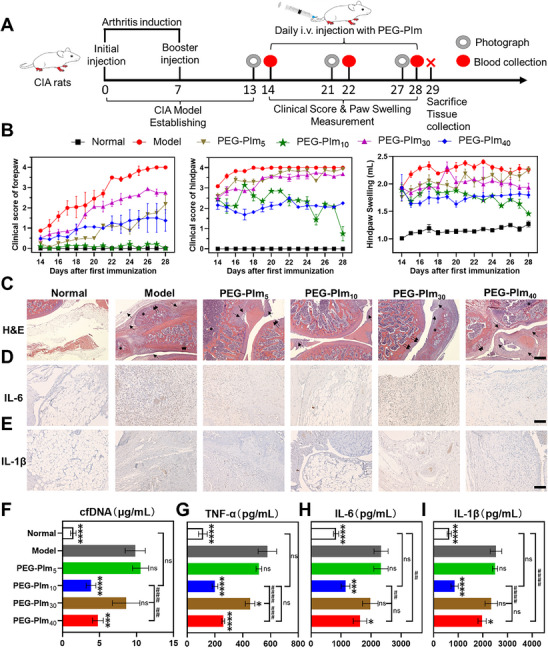
Alleviation of arthritis symptoms of the CIA model treated with PEG‐PIms. A) Experimental timeline of building CIA rats and treating them with PEG‐PIms. After immunizations with type II collagen and Freund's adjuvant twice, the CIA rat model was induced on day 13. From day 13 to day 28, rats received daily intravenous injections of PEG‐PIm solutions. Rats were euthanized on day 29 for further analysis. B) Clinical score of forepaw, hindpaw, and average hindpaw swelling treated from day 13 to day 28. C) Representative H&E staining of knee joints from each group after 15 days of treatment. Infiltration of inflammatory cells in the synovium of CIA rats (single arrow), cartilage erosion (double arrow), and bone destruction (^*^), scale bar: 500 µm. D,E) Immunohistochemical analysis of pro‐inflammatory factors involved in synovial pathology in the knee joints of rats, including IL‐6 and IL‐1β, scale bar: 100 µm. F–I) Changes in levels of cfDNA (F) and pro‐inflammatory factors TNF‐α (G), IL‐1β (H), and IL‐6 (I) in the knees of PEG‐PIm_5_, PEG‐PIm_10_, PEG‐PIm_30_, and PEG‐PIm_40_ treatment groups on day 28 (*n* = 5, mean ± S.E.M.; ^*^0.01 < *p* < 0.05, ^**^0.001 < *p* < 0.01, ^***^0.0001 < *p* < 0.001, ^****^ *p* < 0.0001 compared to the Model group; ^#^ 0.01 < *p* < 0.05, ^##^ 0.001 < *p* < 0.01, ^###^0.0001 < *p* < 0.001, ^####^ *p* < 0.0001 between groups).

As shown above, PEG‐PIm_10_ efficiently degraded the DNAs of NETs and alleviated pulmonary fibrosis in mice model through aerosol inhalation. To prove the efficacy in other NET‐DNA‐induced disorders and expand the application scenario of PEG‐PIms, we established a collagen‐induced arthritis (CIA) rat model, a classical autoimmune disease characterized by elevated extracellular nucleic acids, to explore nucleic acid degradation efficacy by PEG‐PIms following systemic administration (**Figure** **7**A). After injecting PEG‐PIms intravenously, the fluorescent PEG‐PIms mainly accumulated in the articular joints and remained in the joints for up to 24 h and PEG‐PIm_40_ exhibited a relatively longer retention time (Figure S11, Supporting Information). Figure 7B shows the clinical score of the fore‐ and hindpaws of model rats during treatments. Like the therapeutic results observed in lung fibrosis mice via inhalation administration, PEG‐PIm_10_ exhibited a significant inhibitory effect on articular inflammation, whereas PEG‐PIm_40_ followed. As a direct symptom, the swelling volume of hindpaws in the PBS group increased from 1.9 to 2.4 mL. Whereas, in the PEG‐PIm_10_ group, the swelling volume of hindpaws decreased from 1.9 to 1.4 mL. Furthermore, the H&E‐stained results showed that PEG‐PIm_10_ and PEG‐PIm_40_ reduced cartilage and bone destruction compared with the model group as indicated by the black arrows (Figure 7C). The immunohistochemical staining of IL‐6 and IL‐1β on knee joints showed that PEG‐PIm_10_ and PEG‐PIm_40_ obviously downregulated their expression, which effectively alleviated the synovial inflammation (Figure 7D,E).

Subsequently, extracellular DNA and cytokine levels in the synovial tissues were evaluated to support the therapeutic effects. The concentration of the DNA markedly declined after treatment with PEG‐PIm_10_ (3.86 µg mL^−1^) and PEG‐PIm_40_ (4.78 µg mL^−1^) compared to the model group (9.84 µg mL^−1^), confirming their efficacy in controlling the DNA within the joints of CIA rats (Figure 7F). Meanwhile, levels of pro‐inflammatory cytokines, TNF‐α, IL‐6, and IL‐1β, were also significantly reduced in the PEG‐PIm_10_ and PEG‐PIm_40_ groups (Figure 7G–I). Collectively, PEG‐PIm_10_ effectively inhibited the progression of CIA, reduced inflammatory responses and clinical scores, and prevented joint damage in CIA rats.

### 2.7 PEG‐PIms Are Biocompatible to Animal Models

**Figure 8:**
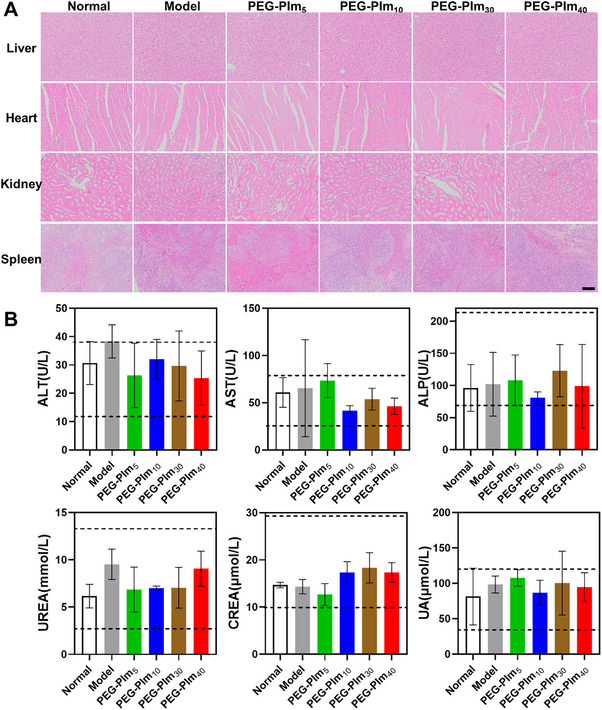
Treatment of PEG‐PIm did not cause any serious lesions of major internal organs. A) HE staining of internal organs including liver, heart, kidney, and spleen of c57 mice after 8 days of inhalation of various PEG‐PIm, scale bar: 100 µm. B) Serum biochemicals including ALP, ALT, AST, creatinine, urea, and uric acid of c57 mice after 8 days of administration of different PEG‐PIm. The normal ranges of ALT, AST, and ALP are 14–38, 28–78, and 67–217 U L^−1^, respectively. The normal ranges of creatinine, urea, and uric acid levels in rat serum are 11–28 µmol L^−1^, 3.2–13.2 mmol L^−1^ and 36–119 µmol L^−1^, respectively. (*n* = 5, means ± S.E.M.).

To explore the biocompatibility of PEG‐PIms, in an anti‐fibrosis experiment involving pulmonary inhalation of PEG‐PIm in C57 mice, major organs and peripheral blood were collected on the 8th day of treatment. Histological analysis using H&E staining was performed on the major organs of the mice (**Figure** **8**A). Blood samples were centrifuged at 300 g for 20 min to obtain cell‐free serum, and the levels of serum biochemical indicators, including alkaline phosphatase (ALP), alanine transaminase (ALT), aspartate transaminase (AST), creatinine (Crea), urea, and uric acid (UA), were analyzed (Figure 8B). ALT, AST, and ALP are key indicators of liver function. Elevated levels of ALT and AST are typically indicative of liver damage, whereas ALP primarily reflects the health of the biliary system. In contrast, creatinine and urea are key markers for assessing kidney function, with increased levels often signifying renal impairment or dysfunction. Furthermore, elevated uric acid levels are commonly associated with metabolic disorders and are also linked to kidney function. The results shown in Figure 8A,B indicated that all data were located within the safe regions, indicating no significant damage to the organs of mice following pulmonary nebulization of PEG‐PIm. Moreover, all the PEG‐PIms were also proven safe in the treatment of collagen‐induced arthritis (CIA) rats via systemic administration (Figure S10, Supporting Information). Thus, the polymeric artificial DNase is safe for further clinical development.

## 3 Discussion

NETosis occurs in response to infection or inflammation and plays an important role in the host's defense against pathogen infection. However, abnormally high levels of NETs are also the main pathogenic factor for pulmonary fibrosis,^[^ ^6^, ^10^ ^]^ cancer metastasis,^[^ ^11^ ^]^ autoimmune diseases,^[^ ^12^ ^]^ and other disorders. For example, it is reported that PF occurs in approximately 20% of COVID‐19 patients.^[^ ^1^ ^]^ Until now, there is still no effective therapeutic strategy for the anti‐fibrotic process. The released DNAs and proteins like myeloperoxidase and neutrophil elastase form NETs by physical interactions. Natural deoxyribonuclease, which can hydrolyze DNA and dissolve the NETs, has been approved for clinical use in patients with cystic fibrosis.^[^ ^13^ ^]^ This strategy might be useful in alleviating mucus secretion in respiratory infection and decreasing the risk of acute respiratory distress syndrome (ARDS). However, these nucleases are often unstable under physiological conditions, and, in this work, we showed they could be dampened by hydrolyzing the nucleic acids within NETs (Figure 2). Recently, we and others applied ionizable polymers as scavengers to block cell‐free DNAs and NET‐DNAs to induce innate immunity and thus inhibit several disorders.^[^ ^14^ ^]^ Thus, targeting NET‐DNAs is a promising strategy in controlling the LF induced by pathogen infection.

Herein, we developed a series of PEGylated‐polyimidazole with tailored composition obtained by controlled polymerization. The base units like imidazole of DNase may attack phosphodiester linkage for a hydrolytic cleavage.^[^ ^15^ ^]^ Since the base units located around the catalytic cavity are synergized in the reaction, the polymer chain bearing multiple imidazole units may accelerate the cleaving process.^[^ ^9b^ ^]^ Thus, the polymeric DNase becomes a good candidate in destroying NETs by degradation of the DNA components.^[^ ^9^ ^]^ It is noteworthy that this work demonstrates that the defined composition is important for cleaving DNA chains. PEG‐PIm is amphiphilic in nature and may self‐assemble into nanoparticles with core/shell structure. As a self‐assembly, there exists a dynamic equilibrium between free polymers and nanoparticles. The imidazole units of free polymer may attack the DNA chain while those of nanoparticles are unlikely to work because of aggregated PIm and shielding PEG shell. The PIm_10_ segment with fewer imidazole units is less hydrophobic and the free chains supply more imidazoles accessible to the phosphodiester linkage. For the samples with longer PIm, it becomes more hydrophobic, and much less free chains are available in equilibrium with nanoparticles. Thus, less effective imidazoles are for accessing the DNA backbones. In terms of PEG‐PIm_5_, though there are many more free chains in media, too fewer imidazole units do not benefit the formation of multiple active sites around the reaction center. This explanation was supported by the MD simulation of the aggregation property of PEG‐PIms.

The potency of cleaving nucleic acids further is demonstrated in weak acidic conditions and in the presence of proteins. The latter case is especially important since the target DNAs form complexes with cationic proteins in NETs. These polymers, especially PEG‐PIm_10_, are positively charged in PBS because of basic imidazole. This allows the PEG‐PIm to destroy NETs and then degrade their DNAs. In contrast, the potency of DNase to degrade NET‐DNAs is dampened in the presence of cationic proteins. Thus, the efficacy of natural DNase in blocking PF induced by NETs could be weakened by proteins like MPO and H3Cit et al, except for concerns of stability and fast clearance.

The NETs induced by respiratory pathogen infection promote lung fibroblasts' transition to myofibroblasts, which express α‐smooth muscle actin and extracellular matrix proteins and generate pulmonary fibrosis. This work shows that this pathway can be blocked by treating with the defined PEG‐PIms that decrease levels of NET‐DNAs in both BALF and infiltration in the lungs, following the hydrolysis efficacy of PEG‐PIms (Figure 5A–C). Furthermore, NET‐DNAs as DMAPs also stimulate pulmonary epithelial and smooth muscle cells via pathogen recognition receptors (PRR) like TLR9, leading to abnormally alleviated cytokines that deteriorate PF. As the results of using PEG‐PIm, the levels of TNF‐α and IL‐6 in BALF are decreased (Figure 5D,E). In terms of symptoms and pathology, the animal models induced by bleomycin significantly benefit from the treatment as shown by the increase of weights, decrease of edema by inflammation, and reduced collagen, namely, pulmonary fibrosis. We evaluated the interventional efficacy through lung coefficient measurement, Masson trichrome staining, and detection of fibrotic‐related factors and NETs markers; however, pulmonary function testing was not incorporated. Therefore, the benefit of treating PF with polymeric DNase is obvious. It is noteworthy that nebulized administration of PEG‐PIm did not enter systemic circulation, significantly escaping the risk in circulation. Furthermore, the concept also works in controlling inflammation of rheumatoid arthritis that is induced by extracellular DNAs, indicating general application in treating extracellular DNA‐induced disorders with polymeric DNase.

## 4 Conclusion

We developed a strategy for hydrolyzing DNAs in NETs utilizing polymer‐based nuclease‐bearing defined imidazole units to inhibit pulmonary fibrosis. We found that the polymers with defined composition may supply more effective imidazole units to cleave the DNA backbones and thus inhibit NETs‐induced migration of primary lung fibroblasts and their transition to myofibroblasts. By aerosol inhalation of the defined polymeric DNase to animal models, the NET accumulation and inflammation in lung as well as pulmonary fibrosis were inhibited. This study highlights the efficacy of artificial nucleases in the control of extracellular‐induced disorders, especially for inflammation‐associated pulmonary fibrosis by NETs. It may be a new strategy to decrease serious lung injury because of some pathogen infection.

## 5 Experimental Section

### Bio‐Reagents

Dulbecco's Modified Eagle Medium (DMEM), Dulbecco's Modified Eagle Medium/Nutrient Mixture F‐12 (DMEM/F12), phosphate buffered solution (PBS), and Fetal Bovine Serum (FBS) were purchased from Gibco. CpG 1826 (sequence: 5′‐TCCATGACGTTCCTGACGTT‐3′) were purchased from Genscript, China and their stock solutions in PBS were prepared as 1 mg mL^−1^. Anti‐alpha smooth muscle Actin (α‐SMA) antibody (cat.#ab5694, Abcam), anti‐Histone H3 (citrullineR2 + R8 + R17) (H3cit) antibody (cat.#ab5103, Abcam), donkey anti‐goat IgG H&L (Alexa Fluor 647) (cat.#ab15031, Abcam), goat anti‐rabbit IgG H&L (Alexa Fluor 488) (cat.#ab150077, Abcam), mouse myeloperoxidase/MPO antibody(cat.#AF3667, R&D), anti‐IL6 antibody (cat.#ab9324, Abcam), anti‐IL‐1β antibody (cat.#ab9722, Abcam), goat anti‐rabbit IgG H&L (HRP) (cat.#ab205718, Abcam), goat anti‐mouse IgG H&L (HRP) (cat.#ab205719,Abcam), phorbol myristate acetate (PMA, Invivogen), DNase I Assay Kit (cat.#ab234056 Abcam), Liberase Research Grade (cat.#5 401 119 001, Roche) were purchased. Ethidium bromide (EtBr) and 3‐(4,5‐dimethyl‐2‐thiazolyl)‐2,5‐diphenyl‐2‐H‐tetrazolium bromide (MTT, purity 98%) were purchased from Sigma Aldrich and their stock solution in PBS was prepared as 1 mg/mL and 5 mg/mL, respectively. To prepare Eosin solution, 2.5 g of Eosin Y (Aladdin, China) was dissolved in 1 L of 95% ethanol with 0.5 mL acetic acid. TRIzol reagent, 4′,6‐diamidino‐2‐phenylindole (DAPI) nucleic acid (NA) stain was purchased from Invitrogen. Mouse TNF‐α ELISA Kit, mouse IL‐6 ELISA Kit, rat TNF‐α ELISA Kit, rat IL‐6 ELISA Kit, and rat IL‐1β ELISA Kit were purchased from Invivogen. Mouse neutrophil extracellular traps (NETs) ELISA Kit (cat. # YS08781B YaJi Biological). Circulating Cell‐free DNA Purification Kit (QIAGEN), immunization grade bovine type II collagen solution (2 mg mL^−1^, Chondrex), complete Freund's Adjust (5 mg/mL, Chondrex), incomplete Freund's Adjust (5 mg/mL, Chondrex), isoflurane (RWD Life Science, China).

### Cells

Primary lung fibroblasts were isolated from male C57 mice following the method described in the reference.^[^ ^16^ ^]^ Cells were cultured in DMEM/F12 medium supplemented with 15% (vol/vol) heat‐inactivated FBS and 100 µg mL^−1^ of the selective antibiotic Zeocin, in a humidified atmosphere at 37 °C with 5% CO_2_. RAW264.7 cells were purchased from ATCC and cultured in DMEM with 10% heat‐inactivated FBS and 100 µg mL^−1^ Zeocin as a selective antibiotic, under the conditions at 37 °C with 5% CO_2_ in a humidified atmosphere.

### Animals

Male C57 mice (6–8 weeks old) were purchased from the Sun Yat‐sen University Experimental Animal Center, and female Wistar rats were obtained from Beijing Vital River Laboratory Animal Technology. All animals were acclimated in the facility for at least 7 days prior to the study. Both rats and mice were housed, maintained, and used under specific pathogen‐free conditions at the Sun Yat‐sen University Animal Experiment Center. All animal studies were approved by the Sun Yat‐sen University Animal Care and Use Committee (Approval Numbers: Rat Study: SYSU‐IACUC‐2022‐001982; Mouse Study: SYSU‐IACUC‐2024‐002555).

### Determination of NA Hydrolysis Activity by PEG‐PIm

The hydrolysis capability of PEG‐PIm at different pH was evaluated using the DNase I Assay Kit, in accordance with the manufacturer's instructions. Briefly, a standard curve of DNA fluorescent product concentration versus absorbance intensity was generated. DNA probes at various concentrations were added to a 96‐well plate, followed by the addition of the DNase I Positive Control from the kit to fully degrade the DNA probes, resulting in a series of DNA probe concentrations post‐hydrolysis. Fluorescence readings were taken using a Multiwell Plate Reader to generate the standard curve of DNA probe fluorescent product concentration versus absorbance intensity. For further analysis, 4 µm of the DNA probe (20 µL per well) was added to each well, followed by 200 µm of PEG‐PIms (20 µL per well). The mixture was incubated at 37 °C for 6 h, after which fluorescence intensity was measured using the Multiwell Plate Reader with an excitation wavelength of 651 nm and an emission wavelength of 681 nm.

### Gel Electrophoresis Analysis of NA Degradation by PEG‐PIm

The method for agarose gel electrophoresis is referenced in this paper.^[^ ^17^ ^]^ PEG‐PIm solutions at different concentrations were incubated with 0.2 µg of CpG in PBS at pH 7.4 and pH 6.5 for 6 h. To exclude potential binding interactions between PEG‐PIm and CpG, NaOH was added to adjust the pH of the mixture to 12 after incubation. The mixture was then loaded onto a 1% agarose gel and electrophoresed at 60 V for 40 min. The gel was imaged using a gel imaging system (Tanon).

In the degradation of DNA in LL37‐CpG immune complexes, 1 µg of LL37 was first incubated with 0.2 µg of CpG for 30 min to ensure complete binding. PEG‐PIm was then added at a PEG‐PIm to CpG mass ratio of 80:1, followed by a 6‐h incubation. The mixture was subjected to gel electrophoresis and subsequently imaged.

Different groups were conducted accordingly.

### Extraction of NETs

Mouse NETs were prepared following the method described in the reference.^[^ ^18^ ^]^ Briefly, neutrophils were isolated using a Mouse Neutrophil Isolation Kit. The collected neutrophils were cultured in RPMI 1640 medium containing 1% BSA and 500 nM PMA at 37 °C with 5% CO_2_ for 4 h. The culture medium was centrifuged at 480 g to pellet the neutrophils, yielding a NET‐rich supernatant. This supernatant was further centrifuged at 18 000 g to isolate the NETs, which were then resuspended in cold PBS. The concentration of the obtained NETs was determined using a PicoGreen dsDNA Quantification Assay Kit.

### Scratch Wound Healing Assay

Cell migration of primary lung fibroblasts under NET stimulation was assessed using a scratch wound healing assay. The extracted primary lung fibroblasts (1 × 10^5^ cells per well) were cultured overnight in a 24‐well plate, and a scratch was created on the cell monolayer using a 200 µL plastic pipette tip. The debris from the scratched cells was gently washed away with PBS, and then 500 µL of serum‐free RPMI 1640 medium was added containing different materials and NETs (Control: serum‐free RPMI 1640; Model: serum‐free RPMI 1640 with 5 µg mL^−1^ NETs; PEG‐PIm: 5 µg mL^−1^ NETs and 400 µg mL^−1^ of four PEG‐PIms dissolved in serum‐free RPMI 1640). Scratch images were captured at 0 h and 24 h using a Leica microscope. The area of the wound closure was measured using ImageJ.

### RNA Extraction and Quantitative RT‐PCR

Primary lung fibroblasts isolated from mice were seeded in 6‐well plates at a density of 3 × 10^6^ cells per well and incubated for 8 h. After this incubation, the medium was replaced with RPMI 1640 containing 10% fetal bovine serum (FBS) and different treatments: Control (RPMI 1640 with 10% FBS), Model (5 µg mL^−1^ NETs), and PEG‐PIm (5 µg mL^−1^ NETs and 400 µg mL^−1^ PEG‐PIms, dissolved in 10% FBS complete medium). Cells were then cultured for an additional 12 h. After incubation, RNA was extracted from the cells using a TRIzol reagent.

For reverse transcription, 2 µg of RNA from each group were converted to cDNA using the PrimeScript RT Reagent Kit with gDNA Eraser and oligo(dT). Quantitative RT‐PCR was performed using TB Green Premix Ex Taq in a LightCycler (Applied Biosystems QPCR system). The mRNA levels of each target gene were normalized to glyceraldehyde‐3‐phosphate dehydrogenase (GAPDH). The relative mRNA expression levels in the control group were set to 1.

The primers used were as follows:

qGAPDH FP: 5′‐TGTGTCCGTCGTGGATCTGA‐3′
qGAPDH AP: 5′‐TTGCTGTTGAAGTCGCAGGAG‐3′
ACTA2: Mouse Acta2 qPCR Primer Pair (cat. # QM09110S, Beyotime).

### Fluorescence Imaging of Primary Lung Fibroblasts

Primary lung fibroblasts were seeded in 8‐chamber confocal dishes and cultured in DMEM/F12 supplemented with 15% fetal bovine serum (FBS) for 8 h. The medium was then replaced with media containing different treatments: Control (DMEM/F12 with 15% FBS), NETs (DMEM/F12 with 15% FBS containing 5 µg mL^−1^ NETs), and PEG‐PIm (DMEM/F12 with 15% FBS containing 5 µg mL^−1^ NETs and 400 µg mL^−1^ of various PEG‐PIms). Cells were cultured for an additional 12 h under the same conditions.

After incubation, the cells were fixed with 4% paraformaldehyde for 15 min and then blocked with 1% bovine serum albumin (BSA) to prevent non‐specific binding. The cells were incubated overnight at 4 °C with the primary antibody anti‐alpha smooth muscle actin (α‐SMA). The following day, a goat anti‐rabbit IgG H&L (Alexa Fluor 488) secondary antibody was added and incubated at 37 °C for 1.5 h. The nuclei were stained with DAPI for 15 min. PBS was used to wash the cells three times after each solution change. Fluorescence images were captured using a Leica confocal microscope, and fluorescence intensity was quantitatively analyzed using ImageJ software.

### C57 Mice Anti‐Fibrosis Experiment

Bleomycin sulfate was dissolved in PBS to prepare a solution with a concentration of 1 mg mL^−1^. This solution was administered via intratracheal injection to the lungs of C57BL/6 mice to establish a fibrosis model. The dosage was 5 mg/kg, with a total injection volume of ≈100 µL (adjusted according to the mouse's body weight). Seven days post‐modeling, a solution of PEG‐PIm was nebulized and inhaled daily into the lungs, while the control group received an equal volume of PBS. Body weight was recorded daily. On day 15, all mice were sacrificed, and bronchoalveolar lavage fluid (BALF) and various tissues were collected for further analysis.

### Histological Staining of Lung

After euthanizing the mice, the lungs were collected and fixed in a 4% paraformaldehyde solution. Following dehydration, the tissue samples were embedded in paraffin, and 2 µm of sections were cut using a microtome (Leica). The tissue sections were stained with the Masson's trichrome staining kit (cat. #G1340, Solarbio). Collagen deposition and lung tissue lesions were assessed using the Vectra automated quantitative pathology imaging system (PerkinElmer).

### Immunofluorescence Staining of Lung

Paraffin‐embedded mouse lung sections were dewaxed and subjected to immunofluorescence staining. Antigen retrieval was performed in 0.01 m of sodium citrate buffer by heating at 125 °C for 30 s, followed by 90 °C for 10 s. After blocking non‐specific binding sites with 10% goat serum in PBS, the sections were incubated with primary antibodies against H3Cit, MPO, and α‐SMA at 4 °C for 24 h. Alexa Fluor 488 and Alexa Fluor 647 conjugated secondary antibodies were then incubated at 37 °C for 1.5 h, followed by DAPI staining for nuclei. After staining, scanning and imaging were performed using a Leica confocal microscope. NETs and α‐SMA fluorescence intensities were quantitatively analyzed using ImageJ software.

### Molecular Dynamics Simulations

Molecular dynamics simulations were employed to explore the self‐assembly behavior of PEG‐PIm polymers in aqueous solutions and the changes in different molecular conformations. First, the ratio of polymers were calculated to be added to the simulation system based on the experimental formulation, as shown in Table S3 (Supporting Information). Then the PEG‐PIm was built and the amber14sb_parmbsc1 forcefield was used in this study.^[^ ^19^ ^]^ The initial system was constructed by randomly inserting individual PEG‐PIm molecules into a cubic box, with a size of 12 × 12 × 12 nm. Afterward, the water molecules were used to solvate this system, and Cl ions were used to neutralize system charges. The system composition information can also be found in Table S3 (Supporting Information). In this study, the steepest descent algorithm was performed to minimize the energy of the system by using the GROMACS 2021.5 software package.^[^ ^20^ ^]^ Then the system was further equilibrated with a temperature thermostat and pressure barostats.^[^ ^21^ ^]^ In brief, the V‐rescale thermostat was used to keep the temperature at 298.15 K, with a coupling time constant of 0.1 ps. The Berendsen barostats were used to control the pressure at 1 bar, with a coupling time constant of 0.5 ps. Then the production simulation ran for at least 100 ns. The periodic boundary conditions were set for all directions. The PMF was applied in electrostatic interactions and the cut‐off was applied in Lennard‐Jones interactions, with a cutoff distance of 1.2 nm. The simulation snapshots were saved every 100 ps. The self‐assembly behavior of PEG‐PIm was visualized by VMD 1.9.2 software^[^ ^22^ ^]^ and The GROMACS homemade analysis tools were used to extract quantitative parameters such as contact number, hydrogen bonds, etc.

### Statistical Analysis

Statistical analysis of all experimental data was performed using one‐way ANOVA followed by the LSD post‐hoc test to assess significant differences between groups. Data were expressed as mean ± standard error of the mean (S.E.M.). The sample size for each experimental group (n) is provided in the figure legends. All statistical tests were two‐sided, and statistical significance was set at α = 0.05, with *p* < 0.05 considered statistically significant. Assumptions of normality and homogeneity of variances were checked using the Shapiro‐Wilk test and Levene's test, respectively. Statistical analyses were performed using GraphPad Prism.

## Conflict of Interest

The authors declare no conflict of interest.

## Author Contributions

Y.B.D. conducted the main experiments and wrote the manuscript; R.W. and D.O.Y. contributed to molecular dynamics simulations; C.Z. contributed to partial cells and animal studies; S.C. and C.L. assisted in partial animal studies; L.L. designed and supervised all the experiments and wrote the manuscript; Y.C. designed materials, supervised, and wrote the manuscript.

## Supporting information



Supporting Information

## Data Availability

The data that support the findings of this study are available from the corresponding author upon reasonable request.
