# Supplementary material for: Inhalable Artificial Polymeric Nucleases Degrading Neutrophil Extracellular Trap‐DNAs and Alleviating Pulmonary Fibrosis
Source: Adv Sci (Weinh). 2025 Jun 20;12(34):e05357. doi: 10.1002/advs.202505357 (PMC12442638; doi:10.1002/advs.202505357)
Supplement: Supplementary file 1 — Supporting Information [file ADVS-12-e05357-s001.docx]

**Supporting information**

**Chemical materials:** 1-(3-aminopropyl) imidazole (J&K Scientific, 98%), methacryloyl chloride (J&K Scientific, 98%), polyethylene glycol (PEG, Aladdin, *M_n_* = 1900 Da), 4-Cyano-4-(dodecylsulfanylcarbonyl) sulfanylpentanoic acid (Aladdin, ≥ 97%), 4-dimethylaminopridine (DMAP, Aladdin, 99%), N, N′-dicyclo-hexyl carbodiimide (DCC, Aladdin, 99%), Alexa Fluor™ 750 C_5_-maleimide (mal-AF750, Thermo Fisher Scientific, A30549), ethanolamine (Aladdin, 99%), N, N-dimethylformamide (DMF, Guangzhou, ≥ 99.7%), dichloromethane (DCM, Guangzhou, ≥ 99.7%), diethyl ether (Guangzhou, ≥ 99.7%) and ethyl acetate (Guangzhou, ≥ 99.7%) were obtained received.

**Instrumentation:** ^1^H NMR spectra were recorded on a Bruker AVANCE III 400 MHz spectrometer with tetramethylsilane (TMS) as the internal standard. Size exclusion chromatography (SEC) was conducted by an Agilent Technologies 1260 Infinity equipped with three PL gel columns (10 μm MIXED-BLS, 5 μm MIXED-C, 5 μm MIXED-D), and a refractive index detector. DMF containing LiBr (0.01 M) was used as an eluent at a flow rate of 1.0 mL/min at 50 ℃. Polystyrene standards were used for calibration. The size and zeta potentials of PEG-PIm were measured using the Zeta-Nanosizer (ZEN3600, Malvern Instruments Ltd, Worcestershire, UK), which was routinely calibrated with a -50 mV Zeta-potential standard (Malvern Instruments). Each measurement was performed for 30 runs, and the results were processed with DTS software version 3.32.

**Synthesis of N-imidazole-3-propylmethacrylamide (ImPMAm):** ImPMAm monomer was synthesized according to the literature^[1]^**.** 10.00 g (0.079 mol) of 1-(3-aminopropyl) imidazole was dissolved in 30 mL of anhydrous DMF, and then 8 mL of DMF solution of 10.45 g (0.1 mol) of methacryloyl chloride was added dropwise at 0 ℃. The reaction mixture was warmed to 30 ℃ and kept stirring for 14 h. Then the reaction was terminated by precipitation into ethyl acetate. The precipitate was dissolved in saturated aqueous sodium bicarbonate solution and stirred until no bubbles generated, following by the extraction with dichloromethane. The organic solution was dried over magnesium sulfate and evaporated to obtain the product ImPMAm, which was verified by ^1^H NMR (Figure S2).

**Synthesis of PEG-CTA:** Polyethylene glycol (PEG) (3.8 g, 2 mmol), 4-Cyano-4-(dodecylsulfanylcarbonyl)sulfanylpentanoic acid (0.612 g, 4 mmol), and 4-dimethylaminopyridine (DMAP) (0.0976 g, 0.8 mmol) were dissolved in dry DCM solution (30 mL). This mixture was added by a DCM solution (10 mL) of N, N′-dicyclo-hexyl carbodiimide (DCC) (0.824 g, 4 mmol) over 30 min. After stirred for 24 h under room temperature, the mixture was filtrated to remove dicyclohexylurea. After the solvent was removed, the residue was precipitated in diethyl ether. The crude was purified by repeated dissolution in CH_2_Cl_2_, precipitated from diethyl ether twice, and finally the pruduct was dried under vacuum at room temperature for 24 h, yield 80%. The structure of PEG-CTA was characterized by ^1^H NMR (Figure S3).

**Synthesis of PEG-PIm:** One sample (PEG-PIm_10_) was supplied. The PEG-PIm polymer was synthesized via reversible addition-fragmentation chain transfer polymerization (RAFT). PEG-CTA (2.81 g, 1.30 mmol), ImPMAm (2.51g, 13mmol), and 10 mL of dried N, N’-dimethylformamide (DMF) were added to a dried polymerization tube and stirred until fully dissolved. The mixture was degassed using three freeze-pump-thaw cycles. Polymerization was carried out at 70 °C for 40 hours and was terminated by cooling and exposure of the solution to air. The reaction mixture was diluted with 10 mL of DMF, followed by the addition of 30 mL of deionized water. The solution was transferred to a dialysis bag (molecular weight cutoff: 1000 Da) and dialyzed against deionized water for 3 days. The product was then obtained by lyophilization. The structure of PEG-PIms were characterized by ^1^H NMR (Figure S4) and SEC (Figure S5, Table S1).

**Fluorescent Labelling of PEG-PIm:** PEG-PIm was dissolved in dried DMF and added to a Schlenk tube. After ethanolamine and mal-AF750 were added, the solution was degassed through two freeze-pump-thaw cycles. Then the reaction was carried out under a nitrogen atmosphere at room temperature and protected from light for 2 hours. The resulting solution was dialyzed in a dialysis bag with molecular weight cutoff 1000 Da against deionized water for 24 hours. The solution of AF750 labelled PEG-PIm was obtained. (Figure S9A)

**Binding Efficiency of PEG-PIm to Nucleic Acids:** The binding efficiency of PEG-PIm was evaluated using the EtBr displacement assay. A mixture of 4 μg of ctDNA and 4 μg of EtBr was prepared in PBS to form the EB-ctDNA complex, to which different volumes of PEG-PIm stock solution and 16 μL FBS were added. After incubation at 37 °C for 6 hours, 100 μL of the supernatant was transferred to a 96-well plate, and the fluorescence intensity of the residual EB-ctDNA complexes was measured using a Multiwell Plate Reader (BioTek Synergy2 Gen5) with an excitation wavelength of 485 nm. The binding efficiency of PEG-PIm and ctDNA was calculated by *(1-(I-I_0_)/(I_1_- I_0_))× 100%*, where *I* was the fluorescence intensity of EtBr-ctDNA complex of supernatant after adding PEG-PIm, *I_0_* was internal EtBr fluorescence intensity, and *I_1_* was the fluorescence intensity of EtBr-ctDNA complexes (Figure S6).

**Cytotoxicity of PEG-PIm:** The cytotoxicity of PEG-PIm was assessed using the MTT assay. Briefly, RAW264.7 cells (3 × 10⁴ cells/well) were plated into a 96-well plate and incubated overnight. The medium was replaced with complete medium containing different concentrations of PEG-PIm, followed by a 24-hour incubation. Then, 10 μL of MTT solution (5 mg/mL) was added to each well and incubated for another 4 hours. The supernatant was removed, and 100 μL of DMSO was added to each well to dissolve the formazan crystals. The absorbance of the solution was measured at 570 nm using a Multiwell Plate Reader (BioTek Synergy2 Gen 5) (Figure S7).

**Induction and Treatment of RA Animal Model:** The RA animal model was established in CIA rats according to literature reference^[2]^. A mixture of bovine type II collagen and Freund's adjuvant was intradermally injected into female Wistar rats on days 1 and 7, with one injection site (0.1 mL) at the tail base and two injection sites (0.2 mL) on the back. On day 13, when arthritis developed, CIA rats were randomly divided into the model group and the treatment group, with five rats in each group. From days 13 to 28, different doses of PEG-PIm were injected daily for treatment, while the model group received PBS as a control. The body weight and toe volume of the rats were recorded daily, and the degree of joint swelling was scored. On day 29, rats from each group were euthanized, and blood, synovial lavage fluid from the knee joint, and various tissues were collected.

**Histological Staining of RA Rat Tissues:** The knee joints of rats sacrificed at different time points were fixed in 4% paraformaldehyde solution. Decalcification was performed at room temperature for 14 days in a decalcification solution (4% hydrochloric acid + 4% formaldehyde). After dehydration and embedding in paraffin, 2 μm sections were cut using a Leica microtome and stained with hematoxylin and eosin. The Vectra automated quantitative pathology imaging system (PerkinElmer) was used to evaluate the infiltration of inflammatory cells in the synovium, cartilage, and bone, as well as the extent of organ lesions.

**Immunohistochemical Staining of Rat Joints:** The paraffin sections of rat knee joints were dewaxed before undergoing immunohistochemical staining. Antigen retrieval was performed in 0.01 M sodium citrate buffer, heating at 125°C for 30 seconds and at 90°C for 10 seconds. Subsequently, the sections were incubated with 3% hydrogen peroxide for 5 minutes to inactivate endogenous peroxidase. After blocking nonspecific binding sites with 10% goat serum in PBS, primary antibodies against IL-6 and IL-1β were incubated at 4°C for 24 hours. The sections were developed using freshly prepared DAB substrate and counterstained with hematoxylin. After dehydration and mounting, the immunohistochemical-stained sections were scanned and imaged using the Vectra automated quantitative pathology imaging system.

**cfDNA Extraction and Quantification from Rat Joint Cavities:** On day 29, each group of Wistar rats was euthanized, and the joint cavities were washed repeatedly with PBS to collect the knee joint lavage fluid. The cfDNA in the lavage fluid was extracted using the Circulating Cell-free DNA Purification Kit and quantified using the Quant-iT^TM^ PicoGreen^TM^ dsDNA assay kit.

**Paw swelling photos:** To evaluate the paw swelling of rats, the phenotypic manifestations of the hind feet of each group were taken by camera on day 13 (1 day before treatment), day 21 (7 days after treatment) and day 27 (13 days after treatment), respectively (Figure S10).

**Sole swelling measurement and clinical score:** From day 14 to day 28, the hind limb swelling of rats in each group was measured by toe volume measuring instrument (YSL-7C, Yiyan Tech, China) every day. Clinical scores of hindlimb and forelimb were given according to the standard rating scale: 0: no evidence of erythema and swelling; 1: erythema and mild swelling occured; 2: erythema and mild swelling extended from ankle to tarsals; 3: erythema and moderate swelling extended from ankle to metatarsal joints; 4: erythema and severe swelling covered ankle, paws, as well as digits or ankylosis of the limbs.


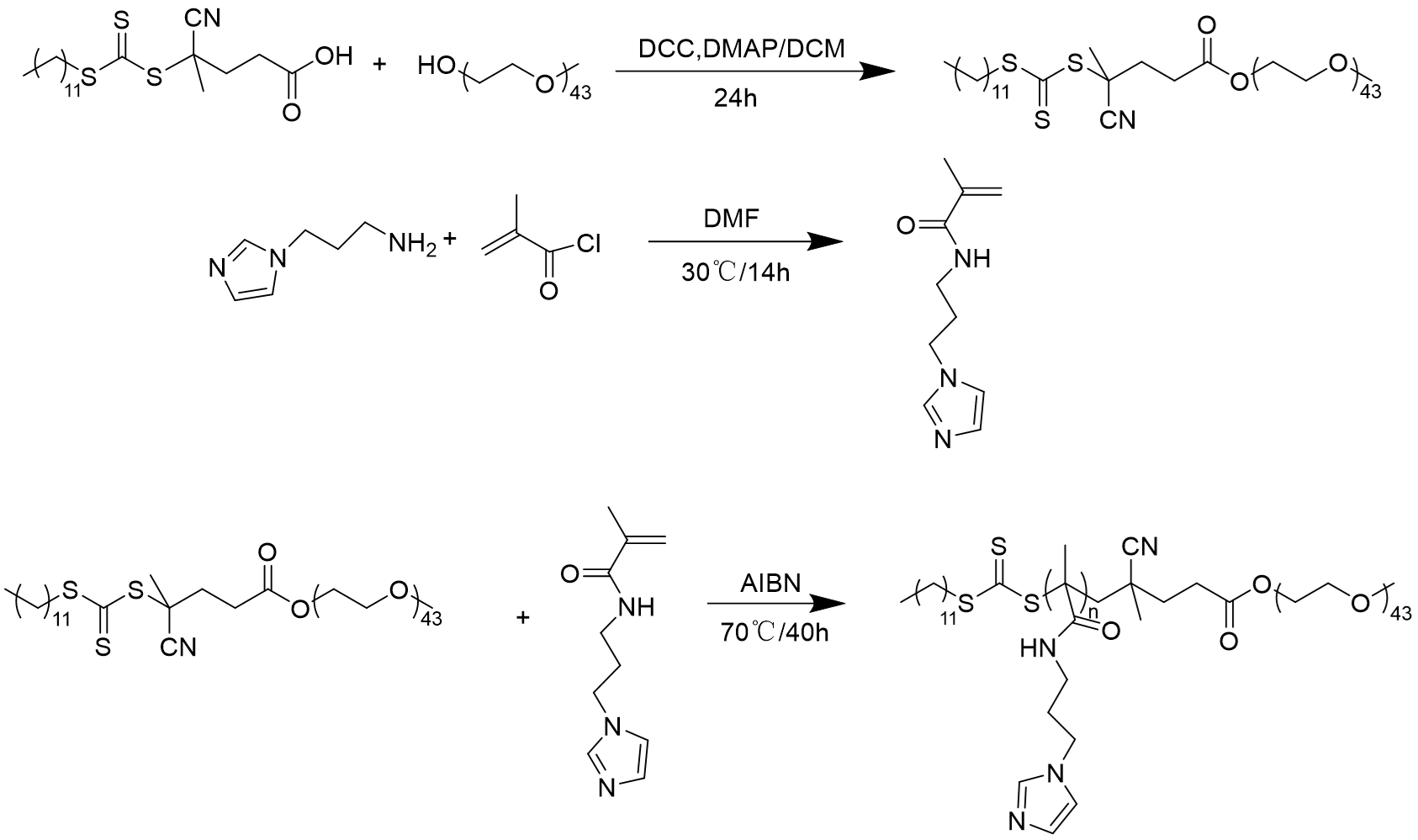


**Figure S1.** Schematic synthesis of PEG initiator, ImPMAm monomer and PEG-PIm block copolymer.


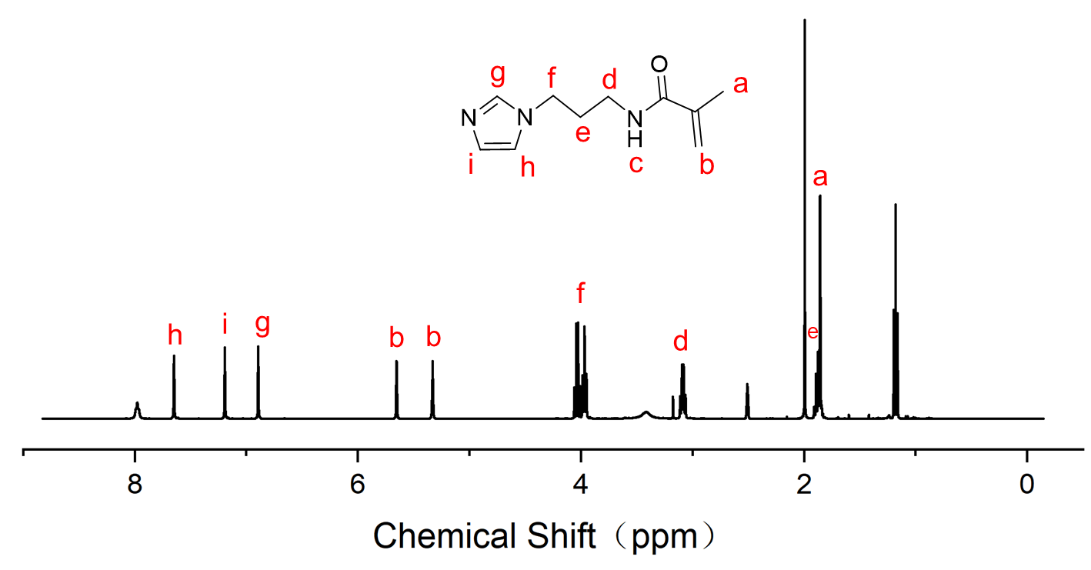


**Figure S2.** ^1^H NMR spectrum of ImPMAm in DMSO-d6 at room temperature.


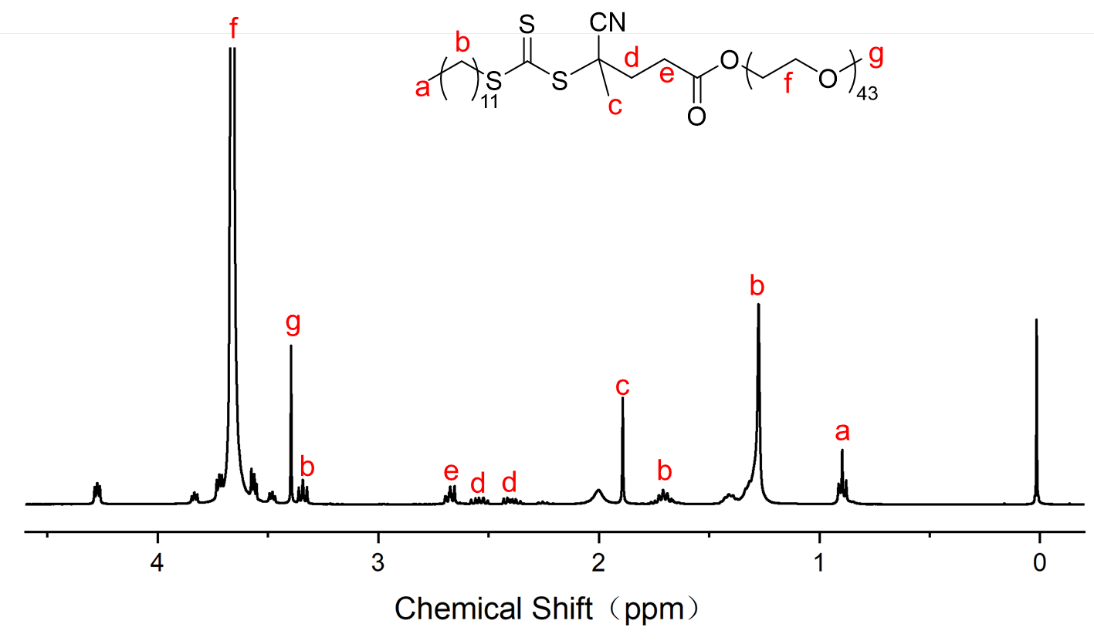


**Figure S3.** ^1^H NMR spectrum of PEG initiator in chloroform-d at room temperature.


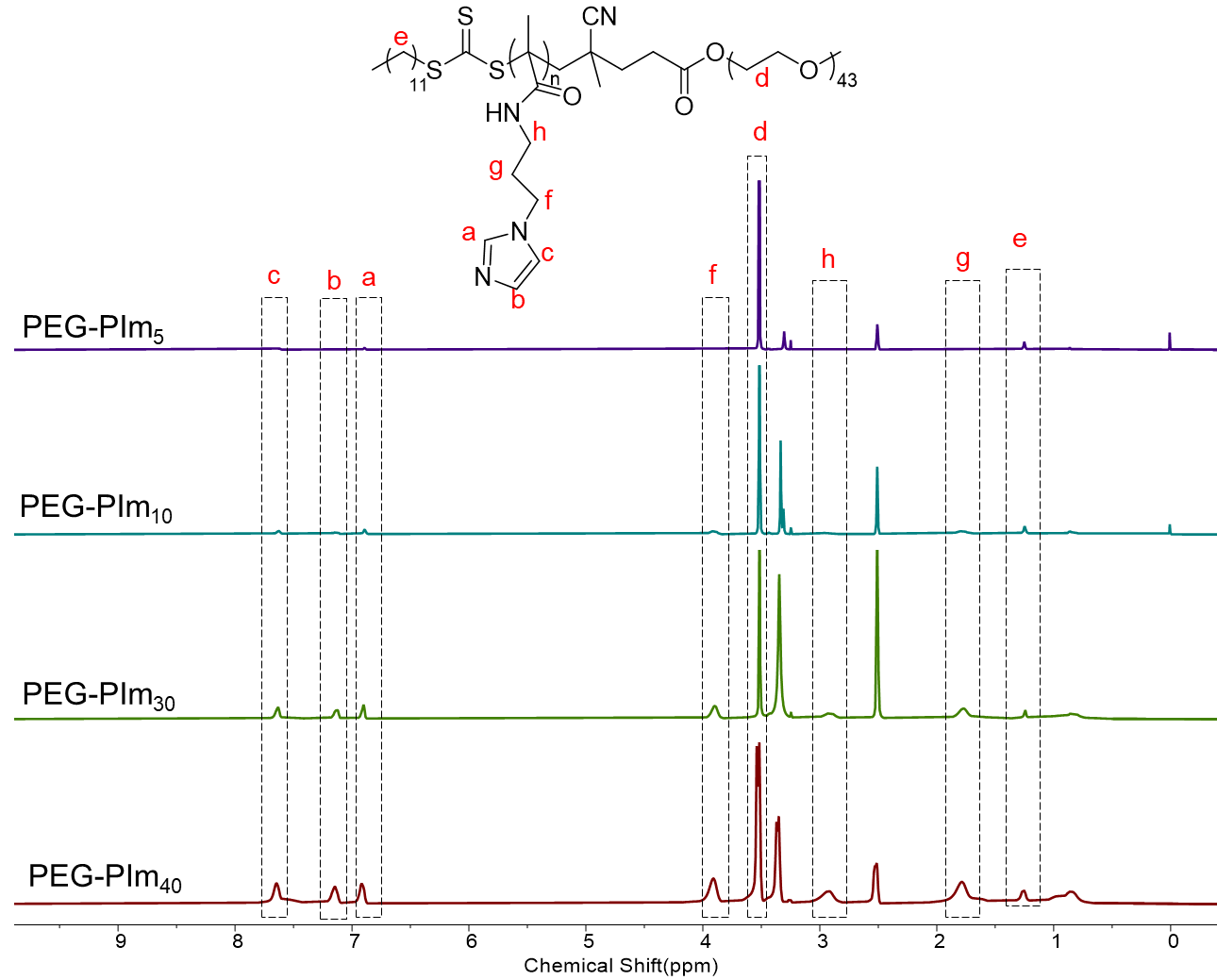


**Figure S4.** ^1^H NMR spectra of PEG-PIm_5_, PEG-PIm_10_, PEG-PIm_30_ and PEG-PIm_40_ in DMSO-d6 at room temperature.


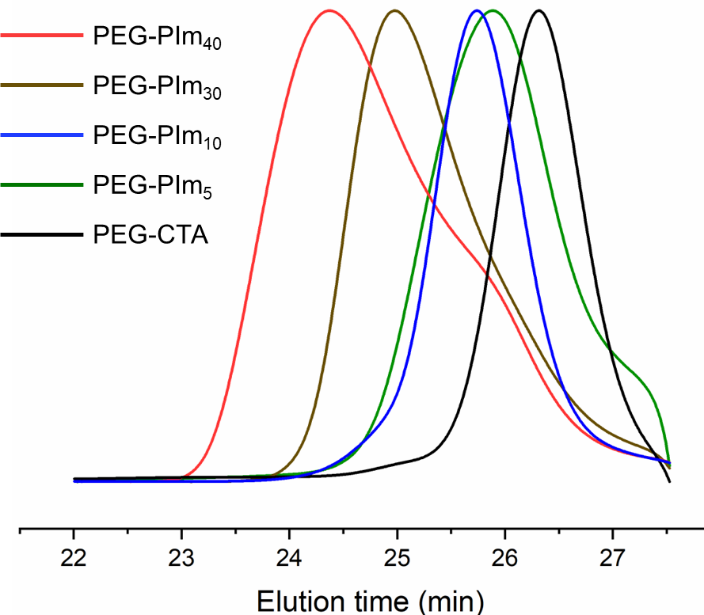


**Figure S5.** SEC traces of PEG-PIm in DMF eluent.


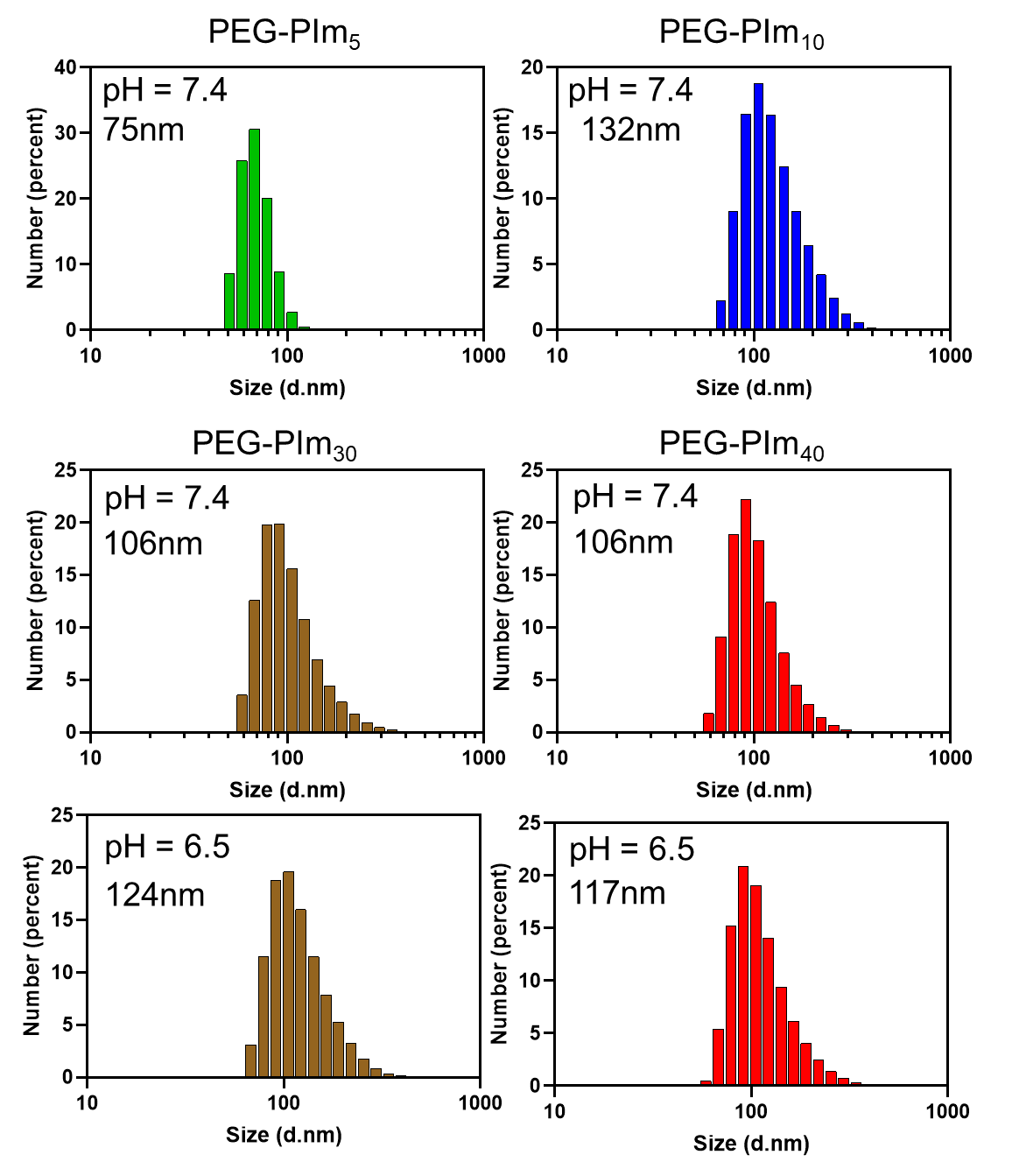


**Figure S6.** Dynamic light scattering (DLS) traces of PEG-PIm_n_ in pH 7.4 and 6.5.


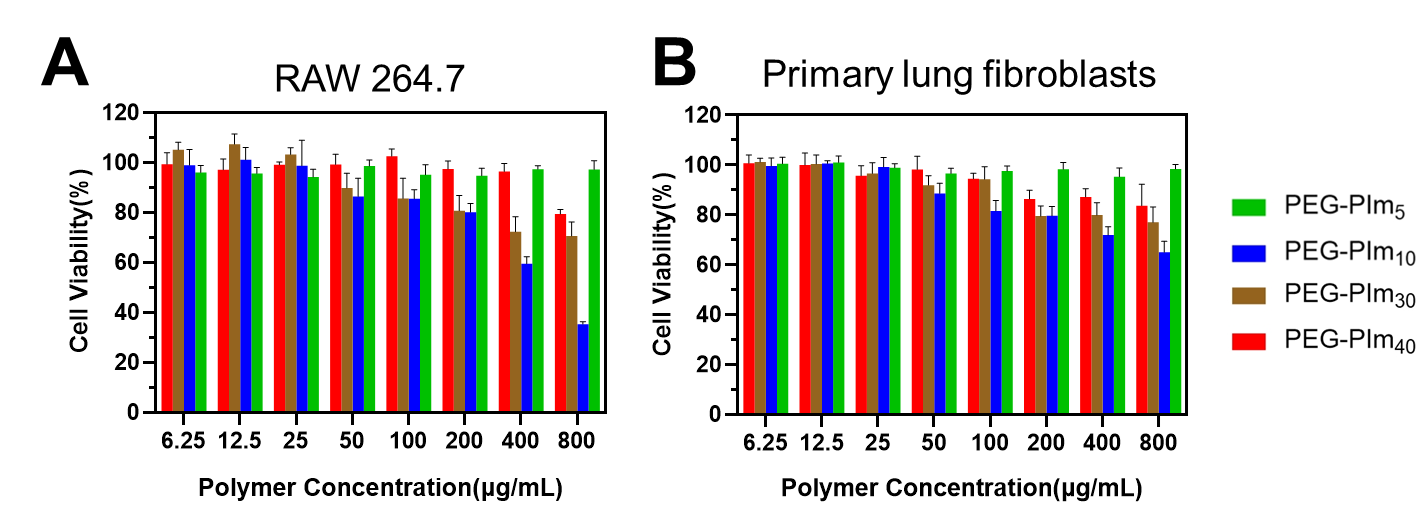


**Figure S7.** Cell viability of RAW 264.7 cells (A) and primary lung fibroblasts (B) treated with different PEG-PIm at various concentrations for 24 h incubation (n = 5, mean ± s.e.m.).


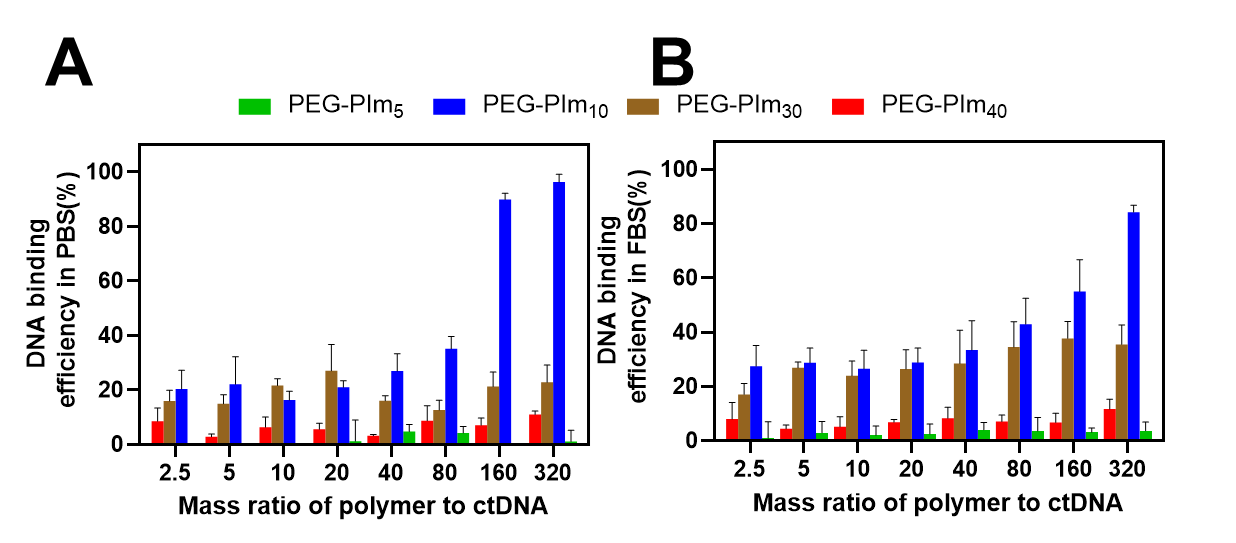


**Figure S8.** Binding efficiency of different PEG-PIm with ctDNAs in **(A)** PBS and **(B)** 10% FBS (n=4, mean ± s.e.m.).


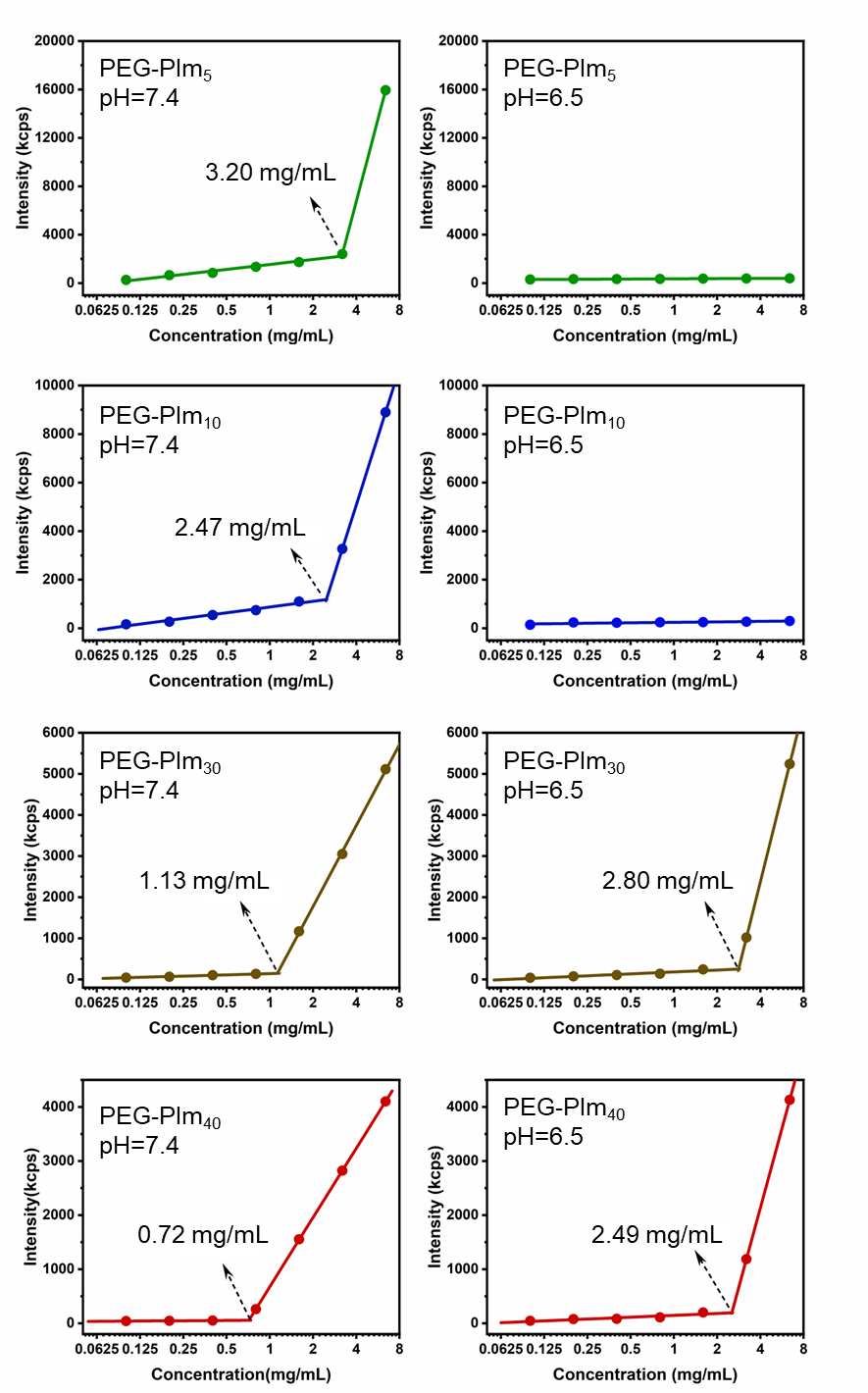


**Figure S9.** Critical micelle concentrations (CMCs) of PEG-PIm_5_, PEG-PIm_10_, PEG-PIm_30_, and PEG-PIm_40_ determined by dynamic light scattering (DLS) at pH 7.4 and 6.5.


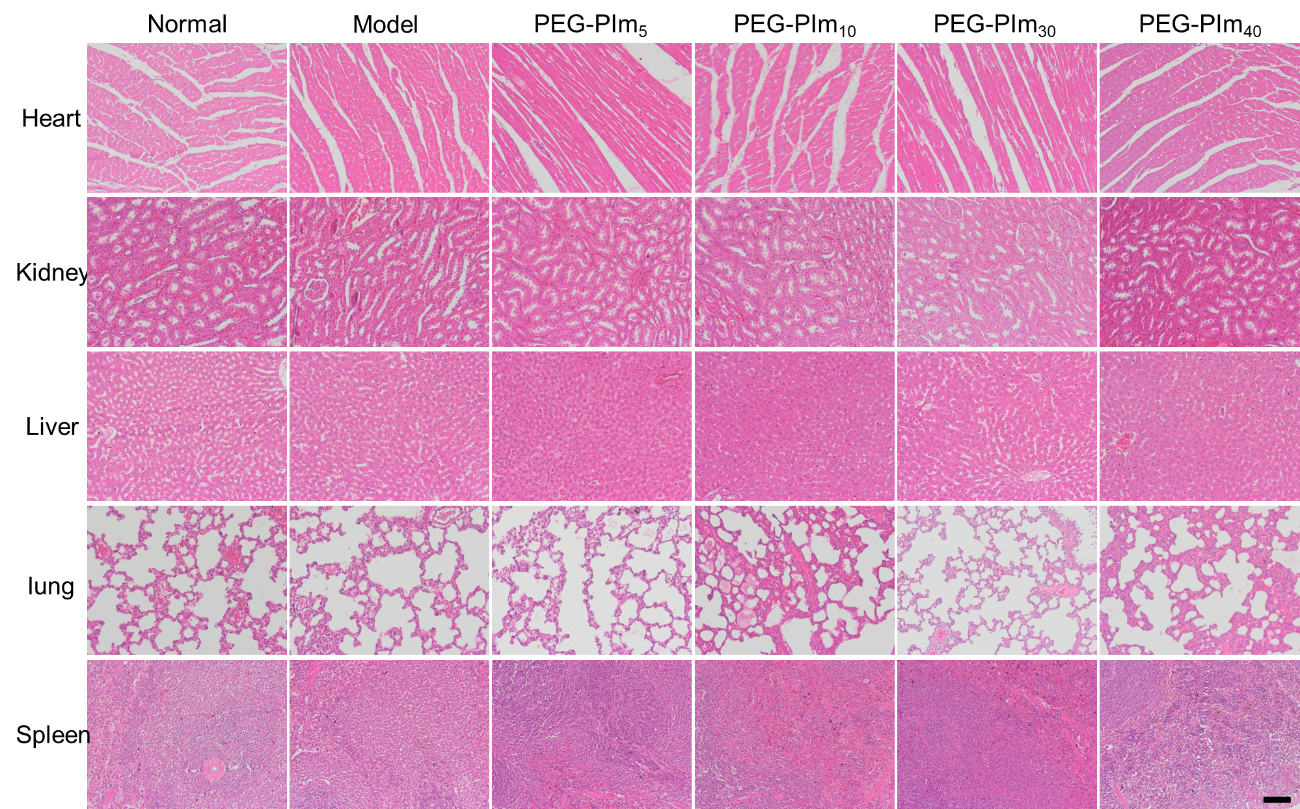


**Figure S10**. HE staining of internal organs including liver, heart, kidney, lung and spleen of CIA rats after 14 days of i.v. injection of PEG-PIm, scale bar: 100 μm.


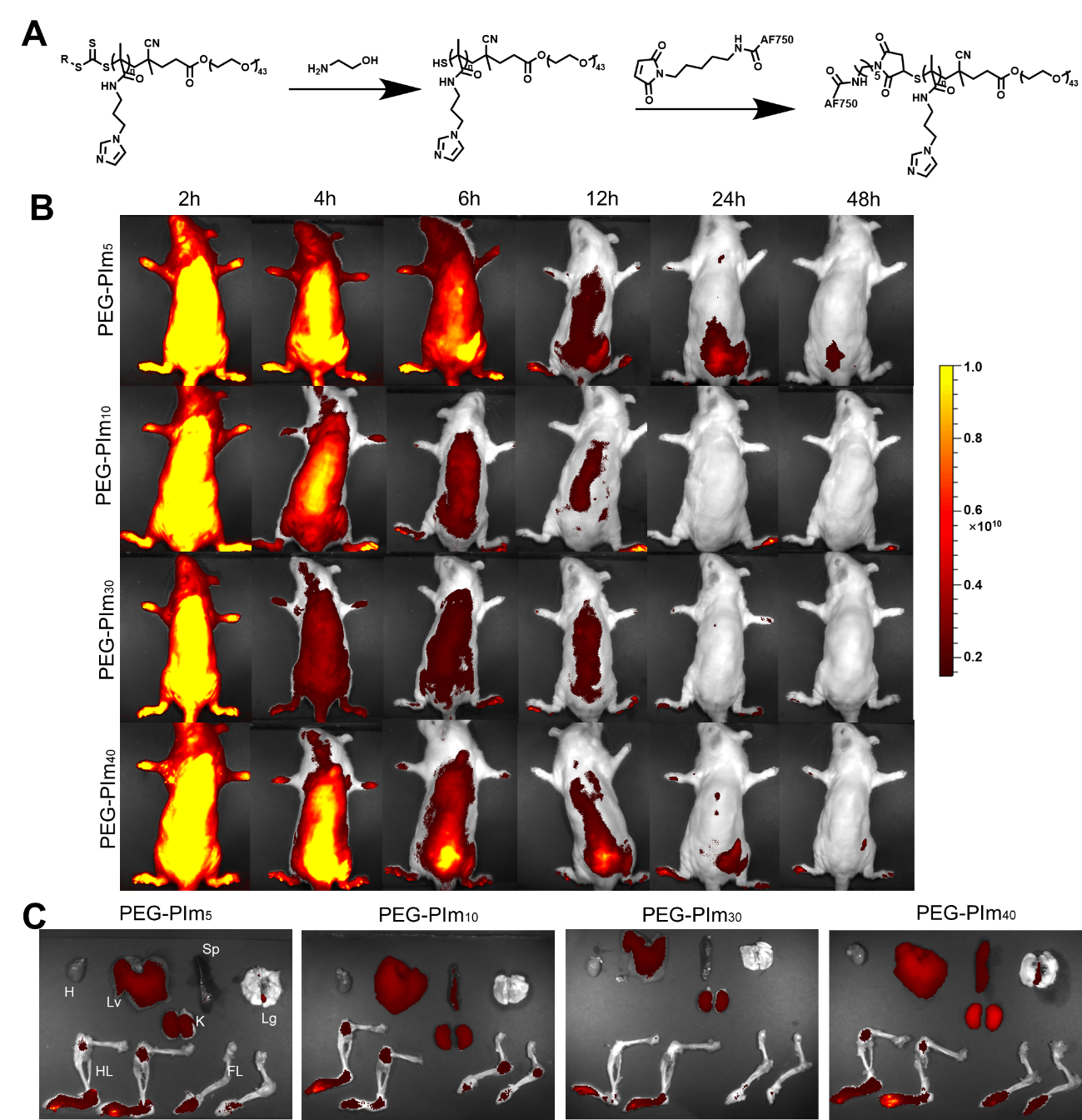


**Figure S11**. Biodistribution of PEG-PIm in CIA rats. **(A)** Schematic synthesis of AF750-PEG-PIm. **(B, C)** Near-infrared fluorescence (NIRF) imaging *in vivo* **(B)** and *ex vivo* **(C)** of CIA rats at various time points after i.v. injection of PEG-PIm. H: heart; Lv: liver; Sp: spleen; Lg: lung; K: kidneys; HL: hindlimbs; FL: forelimbs


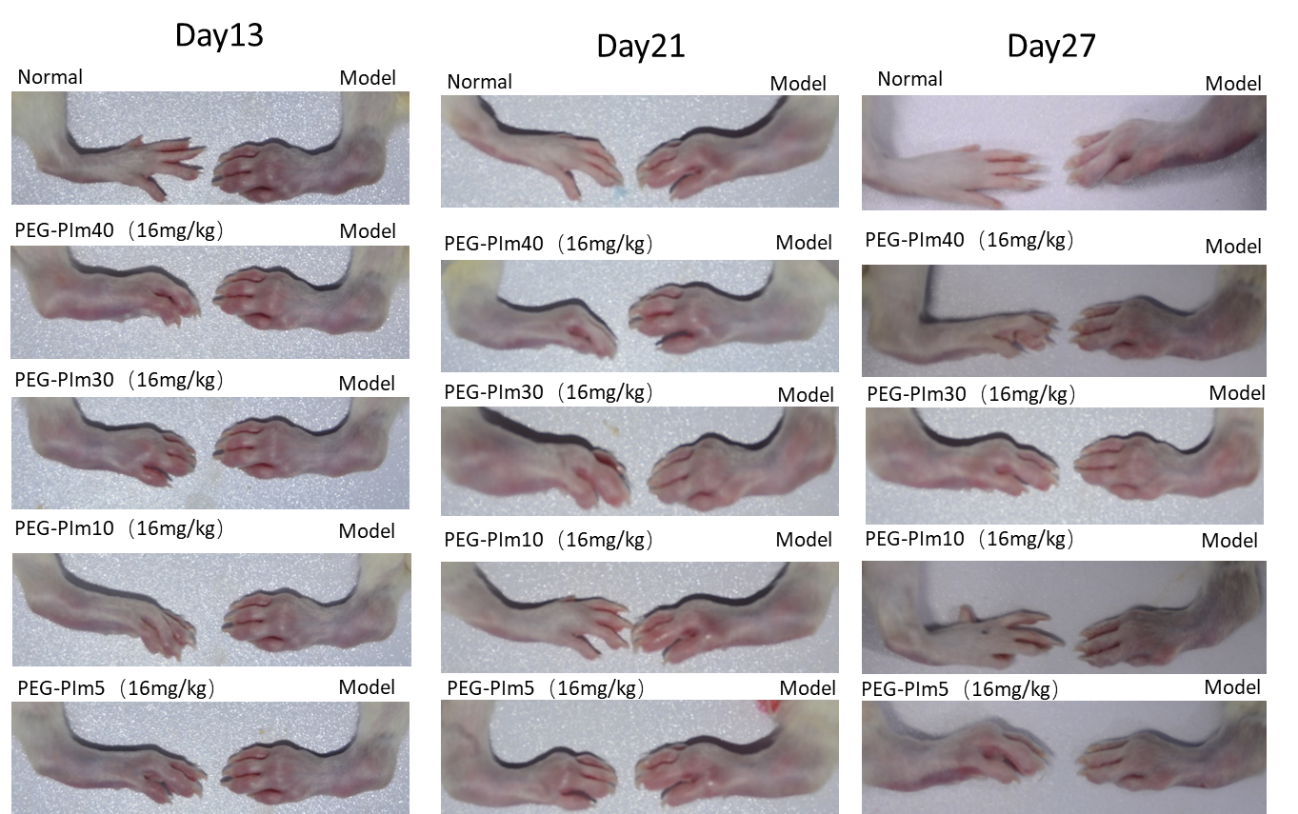


**Figure S12**. Side views of hindpaw swelling of different rats at various time points. Photos of hindpaws were taken on day 13 (1 day before treatment), 21 (7 days after treatment) and 27 (13 days after treatment).

**Table S1. Properties of polymer samples by SEC characterization.**

| Sample | *M_n_* (kDa) | *M_w_/M_n_* |
| --- | --- | --- |
| PEG_43_-PIm_5_ | 3200 | 1.09 |
| PEG_43_-PIm_10_ | 4200 | 1.05 |
| PEG_43_-PIm_30_ | 8100 | 1.08 |
| PEG_43_-PIm_40_ | 10000 | 1.17 |

**Table S2. Particle size and zeta potential of different PEG-PIm in PBS given by dynamic light scattering.**

| Sample | Number Size (nm) | Zeta Potential (mV) |
| --- | --- | --- |
| PEG_43_-PIm_5_ | 75 | 0.94 |
| PEG_43_-PIm_10_ | 132 | 29.5 |
| PEG_43_-PIm_30_ | 106 | 20.2 |
| PEG_43_-PIm_40_ | 106 | 15.8 |

**Table S3. Overview of simulation system composition with neutral PEG-PIm (deprotonation) and charged PEG-PIm (protonation).**

| System | Molecules | Cl^-^ | Water | Total atoms | Box  (nm) | Time  (ns) |
| --- | --- | --- | --- | --- | --- | --- |
| PEG_43_-PIm_10_ (0) | 4 | 0 | 55690 | 169678 | 12×12×12 | 100 |
| PEG_43_-PIm_30_ (0) | 2 | 0 | 55758 | 169738 |  |  |
| PEG_43_-PIm_40_ (0) | 2 | 0 | 55529 | 169631 |  |  |
| PEG_43_-PIm_10_ (+e) | 4 | 28 | 55647 | 169617 |  |  |
| PEG_43_-PIm_30_ (+e) | 2 | 18 | 55719 | 169699 |  |  |
| PEG_43_-PIm_40_ (+e) | 2 | 20 | 55530 | 169734 |  |  |

**SI References**

[1] H. Liang, Y. Du, L. Liu, J. Liu, Y. Chen. A tailored artificial DNase blocks sensor activation and prevents autoimmune and autoinflammatory diseases. Adv. Funct. Mater. **2023**, 33, 2213465.

[2] D. E. Trentham, A. S. Townes, A. H. Kang. Autoimmunity to type II collagen an experimental model of arthritis. J. Exp. Med. **1977**, 146, 857.
